# Highly-sensitive optical organic vapor sensor through polymeric swelling induced variation of fluorescent intensity

**DOI:** 10.1038/s41467-018-06101-8

**Published:** 2018-09-18

**Authors:** Xiangyu Jiang, Hanfei Gao, Xiqi Zhang, Jinhui Pang, Yunqi Li, Kan Li, Yuchen Wu, Shuzhou Li, Jia Zhu, Yen Wei, Lei Jiang

**Affiliations:** 1 CAS Key Laboratory of Bio-inspired Materials and Interfacial Science, Technical Institute of Physics and Chemistry, Chinese Academy of Sciences, 100190 Beijing, China; 2 Engineering Research Center of Special Engineering Plastics Ministry of Education, Jilin University, 130012 Changchun, China; 3 Changchun Institute of Applied Chemistry, Chinese Academy of Sciences, 130022 Changchun, China; 4 School of Material Science and Engineering, Nanyang Technological University, Singapore, 639798 Singapore; 5 Department of Chemistry, Beijing Normal University, 100875 Beijing, China; 6 The Key Laboratory of Bioorganic Phosphorus Chemistry & Chemical Biology, Department of Chemistry, Tsinghua University, 100084 Beijing, China; 7 Beijing Advanced Innovation Center for Biomedical Engineering, Beihang University, 100191 Beijing, China

## Abstract

Traditional optical organic vapor sensors with solvatochromic shift mechanisms have lower sensitivity due to weak intermolecular interactions. Here, we report a general strategy to prepare a higher sensitivity optical organic vapor sensor through polymeric swelling-induced variation of fluorescent intensity. We combine one-dimensional polymeric structures and aggregation-induced emission (AIE) molecules together to form a polymer/AIE microwires array as a sensor. The prepared sensors based on different commercial polymers can successfully classify and identify various organic vapors. Among them, the poly(vinyl butyral)/AIE microwires array can detect methanol vapor as low as 0.05% of its saturation vapor pressure. According to the theory of like dissolves like, we further fabricate a polymer/AIE microwires array derived from designable polyethersulfones, through regulating their side chains, to distinguish similar organic vapors of benzene and toluene. Both experimental and theoretical simulation results reveal that specific molecular interactions between the polyethersulfones and organic vapors can improve the specific recognition performance of the sensors.

## Abstract (web summary)

Traditional optical organic vapor sensors with solvatochromic shift mechanisms have lower sensitivity due to weak intermolecular interactions. Here, the authors report a general strategy to prepare a higher sensitivity optical organic vapor sensor through polymeric swelling-induced variation of fluorescent intensity.

## Introduction

Artificial organic vapor sensor array relays on a finite number of less selective receptors to generate a unique response pattern, yielding responses to a variety of different analytes and providing a fingerprint that allows classification and identification of the analytes^1–5^. Among them, optical sensor arrays provide an efficient and sensitive approach for rapid detection and identification of organic vapor based on colorimetric or fluorescent changes quantified by spectrum^6–8^. In general, an optical sensor array must contain an active center and a chemo-responsive dye, which probes the chemical properties of analytes through strong chemical interactions rather than simple physical adsorption^9,10^. Solvatochromic dye is one of the most important chemo-responsive dyes based on the solvent polarity variation responsible for optical changes^11–15^. Of particular interest for optical organic vapor sensor, the use of polymers doped with a solvatochromic dye (Nile red) as a reporter through polymeric swelling by absorbing analytes has been constructed for optical fiber sensors^16–20^. However, the limitation is that the weak interactions, such as the absorption into polymers or physisorption onto surfaces, usually lead to lower sensitivity of the optical organic vapor sensor. During polymeric swelling, solvatochromic shifts of the fluorescent dyes are typically undetectable below 0.1% of the saturation vapor pressure of an organic solvent^21^. Thus, development of new types of optical organic vapor sensor with alternative mechanism to replace traditional solvatochromic shift type represents an important and highly demanded issue to improve the sensitivity of optical sensor.

Optical organic vapor sensors with aligned one-dimensional (1D) structure have many advantages, such as higher sensitivity, spatial resolution, and rapid response, as compared to those based on films and random fiber networks^22^. The difficulty in manipulating and aligning 1D array hinders the development of high-performance optical sensors. Innovations regarding the superwettability-based interfacial chemistry involved in chemical reaction and nanofabrication have attracted significant attention in energy, environment, and health areas^23–25^. Controllable dewetting process has become an effective strategy to manipulate 1D array formation^26–29^. Aggregation-induced emission (AIE) molecules are nonemissive or provide weak fluorescence in dispersed state, but they become highly fluorescent in aggregated state, which are unique comparing to traditional solvatochromic dyes^30–34^. If we combine 1D polymeric structure and AIE molecule together, polymer/AIE molecule microwires could be fabricated as optical organic vapor sensor. After exposure of polymer/AIE molecule microwires to organic vapor, polymeric swelling occurs and changes the aggregated state of AIE molecules, which will induce variation of fluorescent intensity. In this case, the change of fluorescent signal should be more obvious and sensitive than that of traditional solvatochromic dyes with wavelength shifts. More importantly, regulating the side chains of polymers can introduce specific molecular interaction between polymers and organic vapors, which can provide the opportunity to improve the discrimination and sensitivity of the sensors.

Here, we use a controllable dewetting strategy, termed capillary-bridge-mediated assembly, to fabricate polymer/AIE molecule microwires array as highly sensitive optical organic vapor sensor. The mechanism for organic vapor sensing is through polymeric swelling-induced variation of fluorescent intensity. The prepared microwires array shows good reproducibility and stability upon exposure to organic vapors. Array-based polymer/AIE molecule microwires are fabricated originating from eight commercial polymers, and successfully classify and identify different organic vapors. Through molecular structure regulation of the designed polymers, we further fabricate different types of polymer/AIE molecule microwires array based on six synthetic polyethersulfones with different side chains, to distinguish similar organic vapors of benzene and toluene. We believe this strategy of fabricating polymer/AIE molecule microwires array will provide new route to construct optical organic vapor sensor with high sensitivity.

## Results

### Fabrication of 1D polymer/AIE molecule arrays

A microgroove-structured silicon pillar template is prepared by photolithography and deep-reactive ion etching (DRIE). A selective modification of low-surface-energy perfluoroalkyl silane (FAS) molecule is manipulated onto micropillar sidewalls of the as-prepared template, then yielding the asymmetric-wettability micropillar template with lyophobic sidewalls and lyophilic tops (Detailed descriptions and illustrations of the template fabrication and modification as shown in Supplementary Methods, Supplementary Fig. 1, 2). This asymmetric-wettability micropillar template with the depth of 15 μm is crucial for the directional regulation of liquid fluids, formation of capillary bridges, and controlled deposition of 1D polymer/AIE molecule array.

**Fig. 1:**
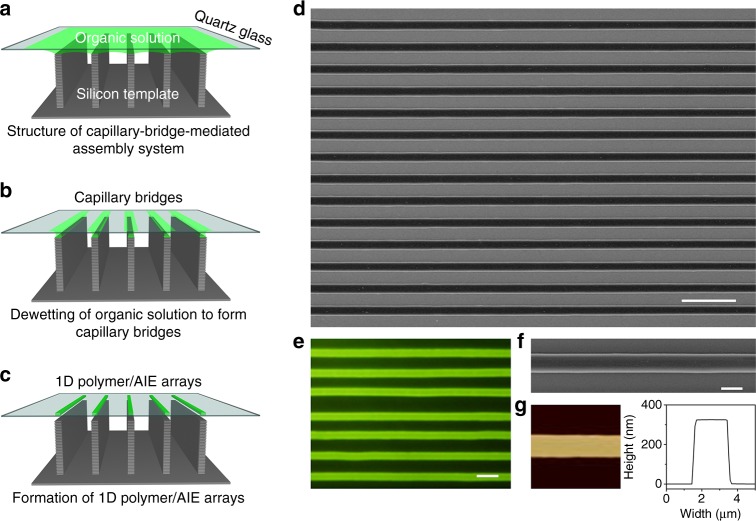
Fabrication of 1D polymer/AIE molecule microwires array. **a**–**c** Scheme showing the capillary-bridge-mediated assembly method for assembly of 1D polymer/AIE molecules microwires arrays. A micropillar-structured template, with lyophobic sidewalls and lyophilic tops, is employed. Then, a thin-film dispersion of polymer/AIE molecule solution is sandwiched between the micropillar template and quartz glass substrate to form the structure of capillary-bridge-mediated assembly system. Dewetted liquid film is gradually divided into capillary bridges, which finally generate 1D polymer/AIE molecule microwires array. **d** SEM image and **e** fluorescent image of large-area 1D polymer/AIE molecule microwires array fabricated on quartz glass. The scale bars are 20 μm and 5 μm for (**d**) and **e**, respectively. **f** Zoom-in SEM image of (**d**), and **g** AFM image showing an individual 1D microstructure with uniform composition, smooth surface, and straight boundary (ca. 2.0 μm in width and ca. 320 nm in height). The scale bar is 2 μm. The PES is employed, and the AIE molecule is AnPh_3_

To fabricate 1D polymer/AIE molecule array, a thin-film dispersion of polymer and AIE molecular solution is sandwiched between the micropillars template and quartz glass to form structure of capillary-bridge-mediated assembly system (Fig. 1a and Supplementary Fig. 3). Owing to the Laplace pressure induced by the asymmetric wettability of micropillars, the three-phase contact line (TCL) is pinned onto tops of micropillars, then leading to the formation of liquid menisci with guiding a vertical dewetting. With development of liquid menisci, the continuous liquid film splits into a series of isolated capillary bridges on the tops of micropillars (Fig. 1b). After further dewetting of capillary bridges, the controlled deposition of the mixture of polymer and AIE molecules was driven by the ordered and directional capillary flows. Finally, 1D polymer/AIE molecule array with uniform composition and precise alignment is fabricated (Fig. 1c). The 1D polymer array fabricated by the capillary-bridge-mediated method performs a high morphological and crystallographic quality (Supplementary Fig. 4 and Note 1).

We employ the scanning electron microscope (SEM), fluorescence microscope, and atom force microscope (AFM) to characterize the morphology and composition of as-fabricated large-area 1D array as shown in Fig. 1d–g. SEM image of 1D array depicts the patterned high-quality microstructures with large area (ca. 160 × 120 μm^2^), precise position and ordered alignment. The fluorescent micrograph shows the strong uniform green fluorescent emission, which illustrates the homodisperse of AIE molecules in the 1D polymer array (Supplementary Fig. 5 and Note 2). A zoom-in SEM image and AFM image exhibit a typical 1D microstructure with smooth surface and straight boundary (ca. 2.0 μm in width and ca. 320 nm in height).

The morphology of micropillar templates is considerable for the fabrication quality of 1D array, and the optimized micropillar template is 5 μm in width and 5 μm in gap. In comparison, 1D polymer/AIE molecule array regulated by the micropillar-structured template with different widths (2 μm and 10 μm) and gaps (2 μm and 10 μm) are formed with irregular and discontinuous morphology (Supplementary Fig. 6). The used polymer is polyethersulfone (PES), and the AIE molecule is 9,10-bis[(*N*-propylphenothiazin-3-yl)vinyl]-anthracene (AnPh_3_). The AIE feature of AnPh_3_ is characterized and shown in Supplementary Fig. 7. The concentration of PES and AnPh_3_ in dimethylformamide (DMF) is 0.30 g L−1 and 0.13 g L^−1^, respectively, and the AnPh_3_ content in the PES/AnPh_3_ microwires is 30%. The diameter of obtained PES/AnPh_3_ microwires is 1.92 μm.

### Proposed mechanism for organic vapor sensing

**Fig. 2:**
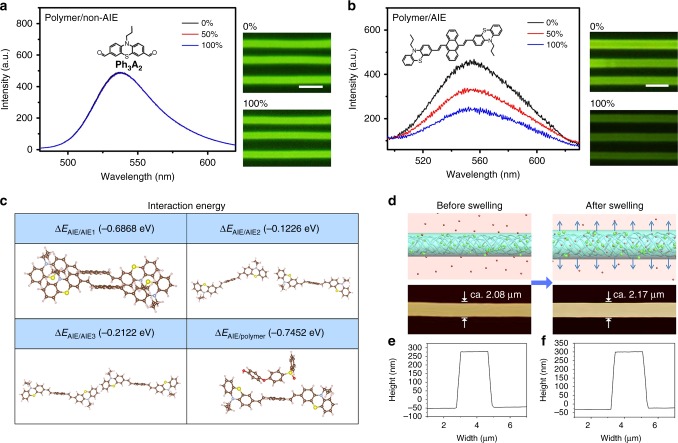
Proposed mechanism of polymer/AIE molecule microwires for organic vapor sensing. Fluorescent emission spectra of **a** PES/non-AIE molecule and **b** PES/AIE molecule microwires before (0%) and after (50 and 100%) exposure to acetone vapor. Inserted photos are fluorescent images of PES/non-AIE molecule and PES/AIE molecule microwires before (0%) and after (100%) exposure to acetone vapor. All scale bars are 5 μm. 100% is the vapor pressure of acetone under ambient condition, and 50% is half of the vapor pressure of acetone under ambient condition. The used polymer is PES, the non-AIE molecule is Ph_3_A_2_, and the AIE molecule is AnPh_3_. The molecular structures of Ph_3_A_2_ and AnPh_3_ are shown as the insets. Both fluorescent excitation wavelengths are 440 nm. **c** Optimized geometry structures and interaction energies of AIE dimers and AIE/polymer for DFT models, proving the stronger interaction of AIE/polymer than that of AIE dimers. **d** The proposed mechanism of fluorescent intensity variation after exposure of polymer/AIE molecule microwires to organic vapor. The absorption of acetone vapor causes swelling of the PES/AnPh_3_ microwires, and increases the intermolecular distance of AnPh_3_ molecules due to the stronger interaction between AnPh_3_ and PES, leading to the dispersion of AnPh_3_ molecules and decreased fluorescent intensity. The used polymer is PES, and AIE molecule is AnPh_3_. The figures below represent AFM topographies of PES/AnPh_3_ microwires before (left) and after (right) exposure to saturated acetone vapor, indicating polymeric swelling increases the width of microwires (by 3.85 ± 0.34%, error bars, s.d. *n* = 20) after exposing the sensor in saturated acetone vapor. **e**, **f** Height diagram of PES/AnPh_3_ microwires, indicating polymeric swelling increases the height of microwires (by 3.31 ± 0.23%, error bars, s.d. *n* = 20) after exposing the sensor in saturated acetone vapor

To prove the feasibility of polymer/AIE molecule microwires array as optical organic vapor sensor, we first investigate the response performance of polymer/non-AIE molecule microwires array to organic vapor (Supplementary Fig. 8 and Note 3). The non-AIE molecule is 10-propyl-phenothiazine-3,7-dicarbaldehyde (Ph_3_A_2_), which is synthesized from *N*-propyl-phenothiazine^35^. The non-AIE feature of Ph_3_A_2_ is characterized and shown in Supplementary Fig. 7. Fluorescent emission spectra of PES/Ph_3_A_2_ microwires before (0%) and after (50 and 100%) exposure to acetone vapor are shown in Fig. 2a, 100% stands for the vapor pressure of acetone under ambient condition, and 50% represents half of the vapor pressure of acetone under ambient condition. The result shows almost no change in the fluorescent intensity of PES/Ph_3_A_2_ microwires after exposure to acetone vapor. The inserted fluorescent images in Fig. 2a also intuitively reveal no response phenomenon of PES/Ph_3_A_2_ microwires when exposed to acetone vapor. In comparison, PES/AnPh_3_ microwires array demonstrates obvious response performance to acetone vapor (Fig. 2b). Under the circumstance of acetone vapor, the fluorescent intensity of PES/AnPh_3_ microwires array decreases to 71% of the original state under half of the saturated vapor pressure of acetone, and 53% of the original state under the saturated vapor pressure of acetone. The inserted fluorescent images in Fig. 2b also indicate the response phenomenon of PES/AnPh_3_ microwires array with variation of fluorescent intensity. This result convinces us that polymer/AIE molecule microwires array possesses obvious response performance to organic vapor.

To investigate the mechanism for organic vapor sensing, density functional theory implemented in the Gaussian 09 package are performed. To determine the interaction energy of AIE dimers and AIE/polymer, density functional theory (DFT) calculations are carried out. Comparing and contrasting the interaction energies, from which the AIE dimers or the isolate AIE molecule and the monomer of polymer, the stability of intermolecular structure can be the logical order of respective models. From the results of AIE dimers and AIE/polymer (Supplementary Table 1, 2 and Fig. 2c), when the functional of wB97XD and B3LYP-D3(BJ) are adopted, the Δ*E*_AIE/polymer_, which are −0.7452 eV and −0.8388 eV, are larger than the interaction energy of AIE dimers. As a consequence, the stronger interaction between AIE and the monomer of polymer is proven, as well as the corresponding structure can be more stable. Moreover, AFM data for the PES/AnPh_3_ microwires before and after exposing the sensor in acetone vapor are provided in Fig. 2d–f. The result evidences the polymer swelling with volume enlargement of the microwires, as the width of microwires increases by 3.85 ± 0.34% and the height increases by 3.31 ± 0.23% after exposing the sensor in acetone vapor. Therefore, the proposed mechanism of the fluorescent intensity variation after exposure of polymer/AIE molecule microwires to the organic vapor is shown in Fig. 2d. The absorption of acetone vapor leads to swelling of the PES/AnPh_3_ microwires^36^, which increases the intermolecular distance of AnPh_3_ molecules due to the stronger interaction between AnPh_3_ and PES, leading to the dispersion of AnPh_3_ molecules and resulting in the decreased fluorescent intensity.

Moreover, we have used four commercial AIE molecules with varied polarity (TPE, TPE-CHO and TPE-OH) and emission (TPE and TPETPAFN) to construct PES/commercial AIE molecule microwires array for organic vapors detection. Fluorescent emission spectra of PES/commercial AIE molecule microwires before (0%) and after (50 and 100%) exposures to acetone vapor are shown in Supplementary Fig. 9, which indicates that all of these commercial AIE molecules can be used for organic vapors detection. In the case of acetone detection, the sensor of PES/TPE microwires has the most obvious relative variations of the fluorescent intensity (Supplementary Fig. 9a). Increasing molecular polarity of AIE molecule or using longer wavelength of AIE molecule has less obvious relative variations of the fluorescent intensity (Supplementary Fig. 9b–d).

### Sensor response characteristics

**Fig. 3:**
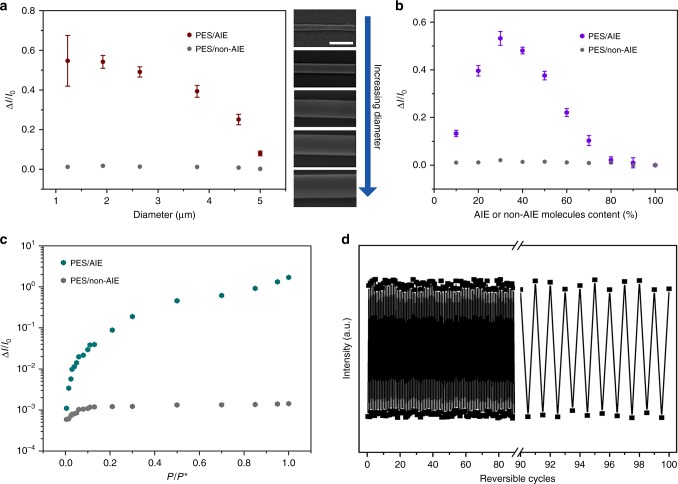
Sensor response characteristics of polymer/AIE molecule microwires. **a** The diameter dependence of the relative variations of the fluorescent intensity, Δ*I*/*I*_0_ (where *I*_0_ is defined as the baseline fluorescent intensity of the PES/AnPh_3_ microwires prior to exposure to acetone vapor, and Δ*I* represents the fluorescent intensity change of these microwires after and before exposure to acetone vapor), shows downward trend with the increase of diameter of the PES/AIE microwires, but shows almost constant in the case of PES/non-AIE microwires. The used polymer is PES, the AIE molecule is AnPh_3_, and the non-AIE molecule is Ph_3_A_2_. Insets are SEM images of PES/AnPh_3_ microwires with different diameters, all scale bars are 5 μm. Error bars, s.d. (*n* = 50). **b** The curve illustrates the dependence of Δ*I*/*I*_0_ on the AIE molecules content or the non-AIE molecules content, which indicates the trend of increasing at first and then decreasing with the increase of AnPh_3_ content of the microwires, but keeps almost constant with the increase of Ph_3_A_2_ content. Error bars, s.d. (*n* = 50). **c** Δ*I*/*I*_0_ for the PES/AnPh_3_ microwires or PES/Ph_3_A_2_ microwires in response to varying partial pressures, *P*, of acetone vapor. P* is the vapor pressure of acetone under ambient conditions. The result indicates that, in the case of PES/AnPh_3_ microwires, Δ*I*/*I*_0_ enhances during increasing the partial pressure of acetone with a nonlinear response at higher acetone concentration, while the Δ*I*/*I*_0_ has a slight increase in the case of PES/ Ph_3_A_2_ microwires. **d** Variations of the fluorescent intensity of the PES/AnPh_3_ microwires upon 100 sequential exposures to acetone vapor, proving good reproducibility and stability of the sensor

To further investigate the sensor response characteristics of PES/AnPh_3_ microwires, we prepare a series of PES/AnPh_3_ microwires with different diameters by controlling the concentration of PES and AnPh_3_ solution. All of AnPh_3_ contents in the PES/AnPh_3_ microwires are designed as 30%. By changing the concentrations of PES in DMF from 0.10 to 1.10 g L^−1^, the diameters of PES/AnPh_3_ microwires increase gradually, demonstrating diameters of 1.23 μm, 1.92 μm, 2.64 μm, 3.77 μm, 4.58 μm, and 5.00 μm for 0.10 g L^−1^, 0.30 g L^−1^, 0.50 g L^−1^, 0.70 g L^−1^, 0.90 g L^−1^, and 1.10 g L^−1^ of the PES concentration, respectively (Supplementary Fig. 10). SEM images of PES/AnPh_3_ microwires with different diameters are shown in the inset of Fig. 3a. The diameter dependence of the relative variations of the fluorescent intensity, Δ*I*/*I*_0_, shows downward trend with the increase of diameter of the microwires, where *I*_0_ is defined as the baseline fluorescent intensity of the PES/AnPh_3_ microwires prior to exposure to acetone vapor, and Δ*I* represents the fluorescent intensity change of these microwires after and before exposure to acetone vapor. In comparison, Δ*I*/*I*_0_ shows almost constant with the increase of diameter of the PES/non-AIE molecule microwires (Non-AIE molecule is Ph_3_A_2_). It should be mentioned that the PES/AnPh_3_ microwires array with a diameter of 1.23 μm has the maximal error bar, and it is found that the microwires array with a diameter of 1.23 μm is inhomogeneous and partially broken (Supplementary Fig. 11a). The fluorescent image of PES/AnPh_3_ microwires is provided to explain the large error bar of the fluorescence of microwires with diameter 1.23 μm (Supplementary Fig. 11b). The discontinuous and broken microwires will lead to decreased fluorescent intensity at the discontinuous area. In contrast, the broken point of the microwires will induce increased fluorescent intensity (Supplementary Fig. 11b, d). These two factors will lead to fluctuations in the fluorescent signal and cause large error bar of the fluorescent intensity. When we tried to fabricate the microwires array with smaller diameter (ca. 0.55 μm), it was found that the microwires had more discontinuous morphologies and severe broken microwires (Supplementary Fig. 11c, d). As the PES/AnPh_3_ microwires array with diameter of 1.92 μm exhibits most obvious relative variations of the fluorescent intensity in response to acetone vapor among these as-prepared PES/AnPh_3_ microwires, it is selected to investigate the sensor response characteristics in detail. Figure 3b shows the dependence of the relative variations of the fluorescent intensity (Δ*I*/*I*_0_) on the AIE molecules content or non-AIE molecules content, which indicates the trend of increasing at first and then decreasing with the increase of AnPh_3_ content of the microwires, but keeps almost constant with the increase of Ph_3_A_2_ content. The maximum value of Δ*I*/*I*_0_ occurs when the AnPh_3_ content of the microwires reaches 30%. Figure 3c shows relative variations of the fluorescent intensity (Δ*I*/*I*_0_) for the PES/AnPh_3_ microwires or PES/Ph_3_A_2_ microwires in response to varying partial pressures, *P*, of acetone vapor. P* is the vapor pressure of acetone under ambient conditions. The exposure times for different concentrations of acetone are sufficient for the microwires to realize maximum fluorescent change. The result indicates that, in the case of PES/AnPh_3_ microwires, the relative variations of the fluorescent intensity are enhanced during increasing the partial pressure of acetone, and exhibits a nonlinear response at higher acetone concentration, indicating the microwires require longer polymer swelling procedure to cross through its percolation threshold. At low acetone vapor concentration, the PES/AnPh_3_ microwires exhibit a good linear dependency between relative variations of the fluorescent intensity and vapor partial pressure. Figure 3c also indicates that the Δ*I*/*I*_0_ has a slight increase during increasing the partial pressure of acetone in the case of PES/Ph_3_A_2_ microwires. Moreover, sensor response time at different concentration of acetone vapor was measured by in situ PL spectrum^37^. The result indicates the sensor response times are 1.89 s, 2.08 s, 2.14 s, and 2.26 s for 25%, 50%, 75%, and 100% of acetone vapor concentration, respectively (Supplementary Fig. 12). Figure 3d shows variations of the fluorescent intensity of the PES/AnPh_3_ microwires upon 100 sequential exposures to acetone vapor, proving good reproducibility and stability for optical organic vapor sensor.

To demonstrate the stability of PES/AnPh_3_ microwires in the presence of water, we test the fluorescent emission and swelling measurements by the immersion in the water fog with different relative humidities (Supplementary Fig. 13). The PES/AnPh_3_ microwires were carefully placed on a sample stage and investigated under different relative humidities (RH) of 25, 50 and 75% (by an ultrasonic humidifier using Milli-Q deionized water). In situ top-view fluorescent microscopy observations were employed under different RH values, which illustrate the same intensity of fluorescent emission and the very small swelling of PES/AnPh_3_ microwires at RH of 25, 50, and 75%. For the quantitative analysis of dependence of fluorescent emission intensity on the environmental RH, we measured in situ photoluminescence spectrum at RH of 25, 50 and 75%, which displays the equivalent intensity of 1D microstructures with RH changing. To further study the resistance of PES/AnPh_3_ microwires to water, AFM is employed for the quantitative characterization of their morphologies in order to infer the swelling property of microwires in water. Typical AFM images of the 1D microstructures (ca. 2 μm in width and ca. 320 nm in height) at RH of 25, 50, and 75% all depict the very small swelling ratio of <2%. According to the measurement and calculation of ten PES/AnPh_3_ microwires, the statistical distribution of their swelling ratios is plotted as shown in Supplementary Fig. 13 (0.83 ± 0.08%, 1.60 ± 0.10%, 1.75 ± 0.07% at RH of 25%, 50%, and 75%, respectively). We suggest that the water adsorption of polymer/AIE molecule tends to be saturated with the increase of environmental RH. Owing to the hydrophobic nature of the nonpolar polymer molecules, the water adsorption of polymer/AIE molecule microwires is very low even though the environmental RH is high. In conclusion, we demonstrate the detection performance of 1D array vapor sensors is not affected by environmental humidity.

### Array-based organic vapors sensing

**Fig. 4:**
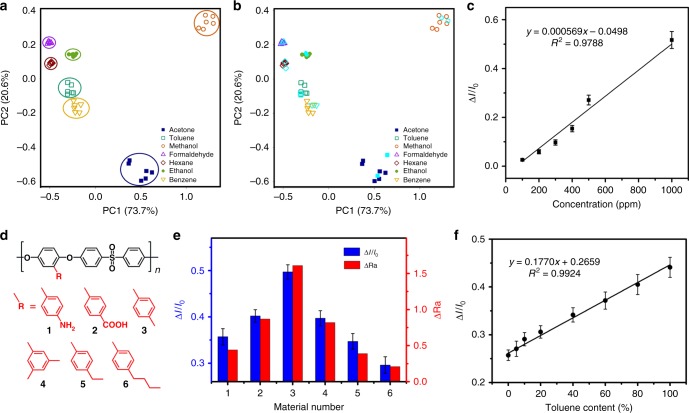
Array-based organic vapors sensing and similar organic vapors identification. **a** PCA plot calculated from the fluorescent intensity variation data obtained for arrays of eight polymer/AnPh_3_ microwires during exposure to seven organic vapors, as presented by the first two principal axes of PC1 and PC2. The used eight commercial polymers include poly(styrene-co-allyl alcohol), poly(α-methylstyrene), poly(vinyl chloride-co-vinyl acetate), poly(vinyl acetate), poly(carbonate bisphenol A), poly(ether sulfone), poly(methyl methacrylate), poly(vinyl butyral), and the AIE molecule is AnPh_3_. The PCA algorithm reveals that 94.3% of the variance in the data can be captured by PC1 and PC2, and shows that different types of the organic vapors are well separated. **b** Verification of the proposed classification method. Another two sets of data (sky-blue color) from these seven organic vapors are measured and evaluated whether the sensors can correctly identify organic vapors through the location of vapors in the PCA plot. The result reveals our proposed method can classify and identify different organic vapors well. **c** Relative variations of the fluorescent intensity (Δ*I*/*I*_0_) for the poly(vinyl butyral)/AnPh_3_ microwires in response to methanol vapor in low concentration, demonstrating a good linear dependency with low limit of detection of 100 ppm. Error bars, s.d. (*n* = 20). **d** The molecular structures of six synthetic PESs with different pendant groups. The pendant groups include 4-aminophenyl, 4-carboxyphenyl, 4-methylphenyl, m-dimethylphenyl, 4-ethylphenyl and 4-butylphenyl groups. **e** Experimental result of benzene and toluene identification with polymer/AnPh_3_ microwires (blue column), and theoretical simulation result of variation of Hansen solubility parameters distance (ΔRa, red column). The used polymer is one of the six synthetic PESs. Experimental result indicates the polymer/AnPh_3_ microwires derived from 4-methylphenyl-containing PES has the best distinction between benzene and toluene. Theoretical simulation result is in good agreement with the experimental result. Error bars, s.d. (*n* = 50). **f** Relative variations of the fluorescent intensity (Δ*I*/*I*_0_) for the polymer/AnPh_3_ microwires derived from 4-methylphenyl-containing PES in response to the mixed benzene/toluene vapor with different toluene content. The result demonstrates a good linear dependency. Error bars, s.d. (*n* = 50)

As each individual PES/AnPh_3_ microwires array has characteristic relative variations of the fluorescent intensity, such data will be useful only in a simple environment such as a single known organic vapor. In order to be applicable to more complex detection, more cross-reactive sensors array will be required to obtain the data. To this end, fluorescent intensity variation data are obtained for arrays of various polymer/AnPh_3_ microwires during exposure to seven organic vapors (Supplementary Fig. 14). In this respect, eight commercial polymers are employed to fabricate different polymer/AnPh_3_ microwires, including poly(styrene-co-allyl alcohol), poly(α-methylstyrene), poly(vinyl chloride-co-vinyl acetate), poly(vinyl acetate), poly(carbonate bisphenol A), poly(ether sulfone), poly(methyl methacrylate), and poly(vinyl butyral). The sensor arrays of these polymer/AnPh_3_ microwires are performed to confirm the classification capability among the following seven organic vapors, including toluene, methanol, formaldehyde, hexane, ethanol, benzene, and acetone. As the detection values of different sensors are highly correlated, principal component analysis (PCA) is used to classify different types of organic vapors^38^. PCA is a statistical method for exploring high-dimensional data sets by reducing the dimensions to the few principal components. Samples can be plotted, making it possible to visually assess similarities and differences between samples and determine whether samples can be grouped. The PCA algorithm reveals that 94.3% of the variation in the data can be captured by the top two principal components, PC1 and PC2, which account for the variances of 73.7% and 20.6%, respectively (Fig. 4a). The PCA result indicates that the top two principal components are enough for the classification, and also shows different types of the organic vapors are well separated. To verify our proposed classification method, we measure another two sets of data from these seven organic vapors, and evaluate whether the sensors can correctly identify the organic vapors through the location of vapors in the PCA plot. We apply the five-nearest estimation based on the top two principal components for classification. Specifically, we first calculate the top two principal components score of the new observations and plot the new observations in the figure. Next, for each new observation, we identify the new observation belongs to the group which has the highest weight in five-nearest points in the training data set. The result is shown in Fig. 4b (plotted in sky-blue color), which demonstrates that our proposed method can classify and identify different organic vapors well, and the classification rate is 100%. Furthermore, four commercial AIE molecules with varied polarity (TPE, TPE-CHO, and TPE-OH) and emission (TPE and TPETPAFN) are used to construct PES/commercial AIE molecule microwires array for array-based organic vapors sensing. PCA plots calculated from the fluorescent intensity variation data obtained for arrays of eight polymer/commercial AIE molecule microwires during exposure to seven organic vapors are provided in Supplementary Fig. 15, indicating different types of the organic vapors are also well separated with these microwires array. This result further verifies the universality of this polymer swelling strategy for commercial AIE molecules. Figure 4c shows relative variations of the fluorescent intensity (Δ*I*/*I*_0_) for the poly(vinyl butyral)/AnPh_3_ microwires in response to low methanol vapor concentration, demonstrating a good linear dependency with low limit of detection of 100 ppm, which equals to 0.05% of the saturation vapor pressure of methanol, revealing the sensing performance is more sensitive than the traditional optical organic vapor sensor with solvatochromic shift mechanism. In addition, the comparative data using a solvatochromic dye (Ph_3_A) to construct PES/Ph_3_A microwires with the same experimental condition, but based on wavelength-shift approach, are provided in Supplementary Fig. 16. The result shows that the fluorescent emission spectra of the PES/Ph_3_A microwires have no wavelength-shift or variation of the fluorescent intensity, which proves the wavelength-shift approach is not applicable to this polymeric swelling method.

### Similar organic vapors identification

Toluene is an important raw material for organic chemical industry that is widely used as solvent and gasoline additive. The current production of toluene is relatively surplus, and a considerable amount of toluene is used for alkylation and disproportionation to produce benzene^39, 40^. Therefore, identification of the toluene content in the mixed toluene/benzene vapors is of great significance. To identify toluene content in the mixed toluene/benzene vapors, we further prepare different types of polymer/AnPh_3_ microwires based on six synthetic polyethersulfones (PESs), according to the theory of like dissolves like. The designed PES polymers contain pendant groups with different side chains, including 4-aminophenyl, 4-carboxyphenyl, 4-methylphenyl, m-dimethylphenyl, 4-ethylphenyl, and 4-butylphenyl groups (Fig. 4d). By regulating the molecular structure of synthetic PES, the as-prepared polymer/AnPh_3_ microwires show obvious distinction for similar organic vapors of toluene and benzene. As shown in Fig. 4e, the polymer/AnPh_3_ microwires array derived from 4-methylphenyl-containing PES has the best distinction between benzene and toluene. In comparison, the polymer/AnPh_3_ microwires array derived from 4-butylphenyl-containing PES exhibits minimum discriminative performance of benzene and toluene. This experimental result proves regulating the side chains of polymers and introducing specific molecular interaction between polymers and organic vapors will improve the discrimination of the sensors. Moreover, theoretical simulation is carried out to investigate the influence of side chains modification of synthetic PESs for the similar organic vapors identification. We conduct Hansen solubility parameters (HSP) combined with the spatial exclusion volume effect of the molecular groups. HSP contain three components: dispersion, polar, and hydrogen bonding. Variations of HSP distance (ΔRa) are calculated to compare the molecular interaction of PES-toluene and PES-benzene. The calculation results indicate the PES/AnPh_3_ microwires array derived from 4-methylphenyl-containing PES has the largest ΔRa, and shows best distinction performance between toluene and benzene vapors. In another word, theoretical simulation result of variation of ΔRa is in good agreement with the relative variations of the fluorescent intensity (Δ*I*/*I*_0_) in the experimental result, which further evidences the theory of like dissolves like is applicable for this strategy. According to Hansen solubility parameter and the theory of like dissolves like, we can easily find the most effective functional groups. From this we know that when our system needs to test a specific organic solvent, it only needs to regulate the side chains of polymers according to the structure of solvent molecules. Finally, we determine relative variations of the fluorescent intensity (Δ*I*/*I*_0_) for the polymer/AnPh_3_ microwires derived from 4-methylphenyl-containing PES polymer in response to the mixed benzene/toluene vapors with different toluene content. The result demonstrates good linear dependency at the whole scope of toluene content (Fig. 4f).

## Discussion

In this work, we prepared polymer/AIE molecule microwires array as highly sensitive optical organic vapor sensor, which adopts a new mechanism of polymeric swelling-induced variation of fluorescent intensity. The prepared optical sensor of PES/AnPh_3_ microwires array achieves the optimal response performance with a diameter of 1.92 μm and the AnPh_3_ content of 30%, and shows good reproducibility and stability upon exposure to organic vapors. We have further fabricated organic vapor sensors from various polymer/AnPh_3_ microwires array originated from eight commercial polymers, and successfully classified and identified seven organic vapors, demonstrating high-specific recognition performance and good separation efficiency according to the PCA result. More importantly, through molecular structure regulation of the designed PESs, we fabricated different types of polymer/AnPh_3_ microwires array based on six synthetic PESs to distinguish similar organic vapors of benzene and toluene. Both experimental and theoretical simulation results prove introducing specific molecular interaction between polymers and organic vapors will improve the discrimination and sensitivity of the sensors. We expect this strategy of fabricating polymer/AIE molecule microwires array will not only open new route to construct novel optical organic vapor sensor with higher sensitivity, but also be used to design heterogeneous optical arrays for liquid sensing.

## Methods

### Fabrication of asymmetric-wettability micropillar template

Silicon wafers (10 cm diameter, N doped, <100> oriented, 525 μm thick) were structured by a photolithography method and deep-reactive ion etching with employing a direct laser writing apparatus, then operated resist stripping for selective modification. First, tops of micropillars were protected by a SU-8 layer. Second, modifiers with low-surface energy were evaporated onto the as-protected templates. Finally, asymmetric-wettability micropillar templates were fabricated after removing the SU-8 layer (See Supplementary Methods).

### Fabrication of PES/AnPh_3_ microwires array

The AIE molecule of AnPh_3_ was synthesized from tetraethyl anthracene-9,10-diylbis(methylene) diphosphonate and 10-propyl-10*H*-phenothiazine-3-carbaldehyde^41^ (See Supplementary Methods). Capillary-bridge-mediated assembly method was used to fabricate polymer/AIE molecule microwires array^42^ (Supplementary Note 4). First, the as-prepared asymmetric-wettability micropillar template was employed. Then, 10 μL dispersion solution of polymer and AIE molecules (dissolved in DMF) is dropped onto the micropillars template and covered by a quartz glass to form the capillary-bridge-mediated assembly system. This assembly system was kept at a temperature of 25 °C and humidity of 60% to control the dewetting in a mild environment. With the evaporation of solvents, the continuous liquid film was divided into a series of capillary bridges by the controlled dewetting. High-quality polymer/AIE molecule microwires were finally generated on the quartz glass by the regulated deposition and molecular packing in the confined space of capillary bridges. All of AnPh_3_ contents in the PES/AnPh_3_ microwires should be equal to 30%. To obtain PES/AnPh_3_ microwires with different diameters, we chose the PES (AnPh_3_) solutions at the concentrations of 0.10 (0.043), 0.30 (0.13), 0.50 (0.21), 0.70 (0.30), 0.90 (0.39), and 1.10 (0.47) g L^−1^.

### Preparation of commercial polymer/AIE microwires array

The procedures of preparing polymer/AIE molecule microwires array with commercial polymers are similar to that of fabrication of PES/AnPh_3_ microwire arrays. The AIE molecule was AnPh_3_. Eight commercial polymers, including poly(styrene-co-allyl alcohol), poly(α-methylstyrene), poly(vinyl chloride-co-vinyl acetate), poly(vinyl acetate), poly(carbonate bisphenol A), poly(ether sulfone), poly(methyl methacrylate), and poly(vinyl butyral) were used in this work. These commercial polymers were dissolved in tetrahydrofuran (THF), acetone, acetone, acetone, acetone, DMF, phenol, and methanol, respectively. The concentrations of these commercial polymers in the corresponding solvent were 0.30 g L^−1^, 0.40 g L^−1^, 0.40 g L^−1^, 0.50 g L^−1^, 0.40 g L^−1^, 0.30 g L^−1^, 0.40 g L^−1^, and 0.25 g L^−1^, respectively. The concentrations of AnPh_3_ were designed as 0.13 g L^−1^, 0.17 g L^−1^, 0.17 g L^−1^, 0.21 g L^−1^, 0.17 g L^−1^, 0.13 g L^−1^, 0.17 g L^−1^, and 0.11 g L^−1^, respectively, to make all of AnPh_3_ contents in the polymer/AIE molecule microwires equal to 30%. The diameters of the polymer/AIE molecule microwires with different commercial polymers were ca. 2 μm.

### Preparation of synthetic PES/AIE microwires array

The procedures of preparing polymer/AIE molecule microwires array with synthetic polyethersulfones are similar to that of fabrication of PES/AnPh_3_ microwires array. The six synthetic polyethersulfones contain different pendant groups, including m-dimethylphenyl, 4-aminophenyl, 4-carboxyphenyl, 4-methylphenyl, 4-ethylphenyl, and 4-butylphenyl groups. The synthetic methods of these six polyethersulfones are shown in the supplementary methods.

### Characterization

A contact angle system (Dataphysics, OCA20, Germany) was employed for the static contact angle measurements of asymmetric-wettability micropillar template, and all angle data were achieved by the measurements in ten different positions of the same sample. The structures of PES/AnPh_3_ microwires array were investigated by scanning electron microscopy (SEM, JEOL, JSM-6700F, Japan) at an accelerating voltage of 3.0 kV. A Nanoscope IIIa instrument (Bruker, ICON2-SYS, Germany) was carried out for AFM measurements of the microwires. To certify the crystallinity of 1D polymer arrays, Grazing incidence wide-angle X-ray scattering (GIWAXS) was performed on XEUSS SAXS/WAXS system. In the system, the incidence angle is 0.2°, and the distance between sample and detector is 122 mm. Fluorescence microscopy images of the polymer/AIE molecule microwires array were obtained using an optical microscope (Vision Engineering Co., UK) coupled to a charge-coupled device (CCD) camera and connected to a desktop computer. The optical measurement was characterized under ambient conditions, using a spectrofluorophotometer (RF-5301-PC; Shimadzu, Kyoto, Japan) with a clean and confined container. For demonstrating the uniform distribution of AIE dye molecules in microwires, a confocal laser scanning microscope (Nikon, N-C2-SIM, Japan) was carried out. The slit widths of both excitation and emission were 5 nm. Proton nuclear magnetic resonance (^1^H NMR) spectra were measured on a JEOL 400 MHz spectrometer, d_6_-DMSO was used as solvent and tetramethylsilane as the internal standard.

### Interaction energy calculation of AIE dimers and AIE/polymer

All calculations were performed using DFT implemented in the Gaussian 09 package^43^. The geometry optimization were performed at B3LYP level using the 6-31G(d, p) basis sets and the single-point energy were calculated at wB97XD/6-31G(d, p) and B3LYP-D3(BJ)/6-31G(d, p) levels, which including dispersion corrections^44^ to obtain the energy of interaction. Due to the skeleton of AIE molecule (AnPh_3_) is similar as AnPh^45^, we used the conformation of AnPh to accelerate the computations. Since there are three different neighbors around an AnPh molecule in AnPh crystal, three AnPh dimers are selected according to AnPh crystal. The geometries of AIE dimers and AIE/polymer were optimized. All structures corresponding to true energy minima were verified by the frequency calculations, which contained only positive frequencies. The single-point energies were then calculated to obtain the energy of interaction, which can be calculated as, Δ*E* = *E*(AB) − *E*(A) − *E*(B) between two molecules A and B in dimers or complex AB^44^, where *E* denotes the single-point energy of respective species. To determine the interaction energy of AIE dimers and AIE/polymer, DFT calculations were carried out. AnPh single-crystal structure is shown in Supplementary Fig. 17, and the optimized geometry structures and interaction energies are shown in Supplementary Table 1, 2.

### Calculation of Hansen solubility parameters distance

The Hansen solubility parameters (HSP) distance between two molecules, conventionally called Ra, is the measure of how alike they are. The famous formula is:

1 $${\rm{Ra}}^2 = 4\left( {\delta {{D}}_1 - \delta {{D}}_2} \right)^2 + \left( {\delta {{P}}_1 - \delta {{P}}_2} \right)^2 + \left( {\delta {{H}}_1 - \delta {{H}}_2} \right)^2$$

The three components are dispersion (*δD*), polar (*δP*), and hydrogen bonding (*δH*). Calculation of variation of Hansen solubility parameters distance (ΔRa) is following the formula below:

2 $$\Delta {\rm{Ra}} = {\rm{Ra}}\left( B \right) - {\rm{Ra}}\left( T \right)$$

Ra(*B*) is the HSP distance between the pendant group in the synthetic PES and benzene, and Ra(*T*) is the HSP distance between the pendant group in the synthetic PES and toluene. Hansen solubility parameters, the value of these parameters is acquired by checking the “Hansen Solubility Parameters: A User’s Handbook”.

## Electronic supplementary material

Supplementary Information

## Data Availability

The authors declare that all the data supporting the findings of this study are available within the paper and its Supplementary Information or from the corresponding author on reasonable request.

## References

[1] Albert KJ (2000). Cross-reactive chemical sensor arrays. Chem. Rev..

[2] Mori K, Nagao H, Yoshihara Y (1999). The olfactory bulb: coding and processing of odor molecule information. Science.

[3] Green E (2006). A rational approach to minimal high-resolution cross-reactive arrays. J. Am. Chem. Soc..

[4] Vosshall LB, Stocker RF (2007). Molecular architecture of smell and taste in Drosophila. Annu. Rev. Neurosci..

[5] Finger TE, Bartel DL, Shultz N, Goodson NB, Greer CA (2017). 5HTR3A-driven GFP labels immature olfactory sensory neurons. J. Comp. Neurol..

[6] Rakow NA, Suslick KS (2000). A colorimetric sensor array for odour visualization. Nature.

[7] Walt DR (2000). Bead-based fiber-optic arrays. Science.

[8] Lin H, Jang M, Suslick KS (2011). Preoxidation for colorimetric sensor array detection of VOCs. J. Am. Chem. Soc..

[9] You L, Zha D, Anslyn EV (2015). Recent advances in supramolecular analytical chemistry using optical sensing. Chem. Rev..

[10] Basabe-Desmonts L, Reinhoudt DN, Crego-Calama M (2007). Design of fluorescent materials for chemical sensing. Chem. Soc. Rev..

[11] Reichardt C (2007). Solvents and solvent effects: an introduction. Org. Process Res. Dev..

[12] Katritzky AR (2004). Quantitative measures of solvent polarity. Chem. Rev..

[13] Stitzel SE, Aernecke MJ, Walt DR (2011). Artificial noses. Annu. Rev. Biomed. Eng..

[14] Buss CE, Mann KR (2002). Synthesis and characterization of Pt(CN-*p*-(C_2_H_5_)C_6_H_4_)_2_(CN)_2_, a crystalline vapoluminescent compound that detects vapor-phase aromatic hydrocarbons. J. Am. Chem. Soc..

[15] Hudson ZM (2011). Probing the structural origins of vapochromism of a triarylboron-functionalized platinum (II) acetylide by optical and multinuclear solid-state NMR spectroscopy. Inorg. Chem..

[16] Dickinson TA, White J, Kauer JS, Walt DR (1996). A chemical-detecting system based on a cross-reactive optical sensor array. Nature.

[17] Walt DR (2010). Fibre optic microarrays. Chem. Soc. Rev..

[18] Levitsky I, Krivoshlykov SG, Grate JW (2001). Rational design of a Nile Red/polymer composite film for fluorescence sensing of organophosphonate vapors using hydrogen bond acidic polymers. Anal. Chem..

[19] Li D, Mills CA, Cooper JM (2003). Microsystems for optical gas sensing incorporating the solvatochromic dye Nile Red. Sens. Actuat. B Chem..

[20] White J, Kauer JS, Dickinson TA, Walt DR (1996). Rapid analyte recognition in a device based on optical sensors and the olfactory system. Anal. Chem..

[21] Askim JR, Mahmoudi M, Suslick KS (2013). Optical sensor arrays for chemical sensing: the optoelectronic nose. Chem. Soc. Rev..

[22] LaFratta CN, Walt DR (2008). Very high density sensing arrays. Chem. Rev..

[23] Wang S, Liu K, Yao X, Jiang L (2015). Bioinspired surfaces with superwettability: new insight on theory, design, and applications. Chem. Rev..

[24] Wen L, Tian Y, Jiang L (2015). Bioinspired super-wettability from fundamental research to practical applications. Angew. Chem. Int. Ed..

[25] Su B, Tian Y, Jiang L (2016). Bioinspired interfaces with superwettability: from materials to chemistry. J. Am. Chem. Soc..

[26] Huang J, Kim F, Tao AR, Connor S, Yang P (2005). Spontaneous formation of nanoparticle stripe patterns through dewetting. Nat. Mater..

[27] Han W, Byun M, Li B, Pang X, Lin Z (2012). A simple route to hierarchically assembled micelles and inorganic nanoparticles. Angew. Chem. Int. Ed..

[28] Van Hameren R (2006). Macroscopic hierarchical surface patterning of porphyrin trimers via self-assembly and dewetting. Science.

[29] Jiang X (2016). Bioinspired 1D superparamagnetic magnetite arrays with magnetic field perception. Adv. Mater..

[30] Li D, Qin W, Xu B, Qian J, Tang BZ (2017). AIE nanoparticles with high stimulated emission depletion efficiency and photobleaching resistance for long-term super-resolution bioimaging. Adv. Mater..

[31] Cheng Y (2017). Multiscale humidity visualization by environmentally sensitive fluorescent molecular rotors. Adv. Mater..

[32] Zhang X (2014). Aggregation induced emission-based fluorescent nanoparticles: fabrication methodologies and biomedical applications. J. Mater. Chem. B.

[33] Wang J (2017). Ionization and anion−π^+^ interaction: a new strategy for structural design of aggregation-induced emission luminogens. J. Am. Chem. Soc..

[34] Peng HQ (2017). Dramatic differences in aggregation-induced emission and supramolecular polymerizability of tetraphenylethene-based stereoisomers. J. Am. Chem. Soc..

[35] Sun X (2005). Novel electroactive and photoactive molecular materials based on conjugated donor−acceptor structures for optoelectronic device applications. J. Phys. Chem. B.

[36] Champeau M (2014). In situ FTIR micro-spectroscopy to investigate polymeric fibers under supercritical carbon dioxide: CO_2_ sorption and swelling measurements. J. Supercrit. Fluids.

[37] Xue P (2017). Aggregation-induced emission nanofiber as a dual sensor for aromatic amine and acid vapor. J. Mater. Chem. C.

[38] Jurs P, Bakken G, McClelland H (2000). Computational methods for the analysis of chemical sensor array data from volatile analytes. Chem. Rev..

[39] Kubů M (2016). Three-dimensional 10-ring zeolites: the activities in toluene alkylation and disproportionation. Catal. Today.

[40] Gallego EM (2017). “Ab initio” synthesis of zeolites for preestablished catalytic reactions. Science.

[41] Zhang X (2014). Influence of alkyl length on properties of piezofluorochromic aggregation induced emission compounds derived from 9, 10-bis[(*N*-alkylphenothiazin-3-yl)vinyl]anthracene. Tetrahedron.

[42] Feng J (2017). “Capillary-bridge lithography” for patterning organic crystals toward mode-tunable microlaser arrays. Adv. Mater..

[43] Frisch MJ (2009). Gaussian 09, Revision A.1.

[44] Grimme S (2016). Dispersion-corrected mean-field electronic structure methods. Chem. Rev..

[45] Zhang X (2011). Piezofluorochromic properties and mechanism of an aggregation-induced emission enhancement compound containing *N*-hexyl-phenothiazine and anthracene moieties. J. Phys. Chem. B.

