# Supplementary material for: Highly-sensitive optical organic vapor sensor through polymeric swelling induced variation of fluorescent intensity
Source: Nat Commun. 2018 Sep 18;9:3799. doi: 10.1038/s41467-018-06101-8 (PMC6143602; doi:10.1038/s41467-018-06101-8)
Supplement: Supplementary file 1 — Supplementary Information [file 41467_2018_6101_MOESM1_ESM.pdf]

Supplementary Information

Highly-sensitive optical organic vapor sensor through polymeric swelling induced variation of fluorescent intensity

Xiangyu Jiang[1], Hanfei Gao[1], Xiqi Zhang[1,*], Jinhui Pang[3], Yunqi Li[4], Kan Li[1], Yuchen Wu[1,*], Shuzhou Li[7], Jia Zhu[6], Yen Wei[5], Lei Jiang[1,2]

[1] CAS Key Laboratory of Bio-inspired Materials and Interfacial Science, Technical Institute of Physics and Chemistry, Chinese Academy of Sciences, Beijing 100190, P. R. China

[2] Beijing Advanced Innovation Center for Biomedical Engineering, Beihang University, Beijing 100191, P. R. China

[3] Engineering Research Center of Special Engineering Plastics Ministry of Education, Jilin University, Changchun 130012, P. R. China

[4] Changchun Institute of Applied Chemistry, Chinese Academy of Sciences, Changchun 130022, P. R. China

[5] The Key Laboratory of Bioorganic Phosphorus Chemistry & Chemical Biology, Department of Chemistry, Tsinghua University, Beijing 100084, P. R. China

[6] Department of Chemistry, Beijing Normal University, Beijing 100875, P. R. China

[7] School of Material Science and Engineering, Nanyang Technological University, Singapore 639798, Singapore

* Correspondence and requests for materials should be addressed to X. Q. Z., Y. C. W. (Emails: xqzhang@mail.ipc.ac.cn, wuyuchen@iccas.ac.cn)

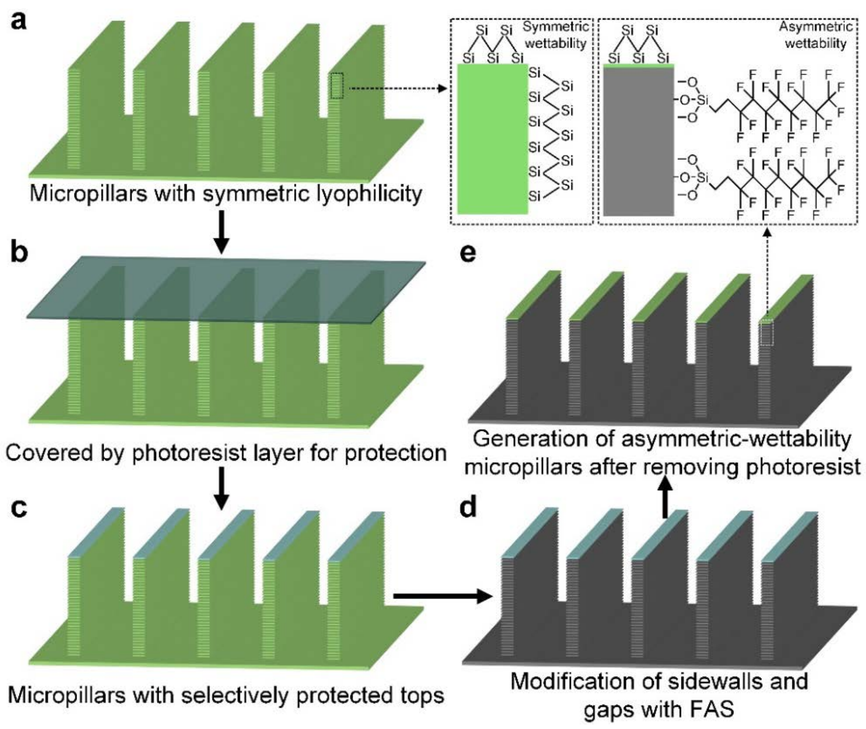

Supplementary Figure 1. Asymmetric modification of micropillar-structured template. (a) The symmetric-wettability micropillar template with lyophilic tops and sidewalls. (b) For the asymmetric modification of template, a flat substrate with a spin-coated SU-8 layer was loaded onto the template. (c) Tops of micropillars were selectively protected after peeling off the substrate and baking. (d) FAS molecules were modified onto the top-protected templates. (e) After removing the SU-8 protection layer, micropillar templates with lyophilic tops and lyophobic sidewalls were fabricated.

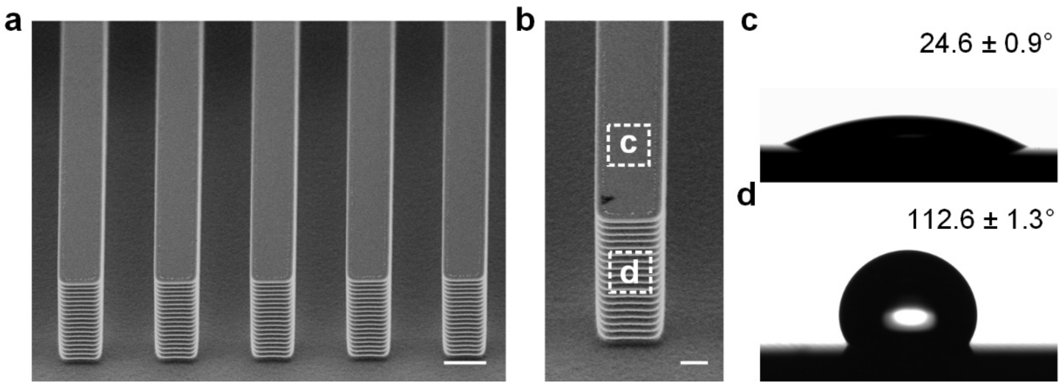

Supplementary Figure 2. Morphology of micropillar-structured template. (a) SEM image and (b) magnified SEM image of the micropillar-structured template in an overlooking manner with the width of 5 μm, separation of 5 μm, and depth of 15 μm. Scale bars are 5 μm and 2 μm for (a) and (b), respectively. (c) A low contact angle of 24.6 ± 0.9° at the top and (d) high contact angle of 112.6 ± 1.3° at the sidewall of micropillars. Error bar, s.d. (n = 10).

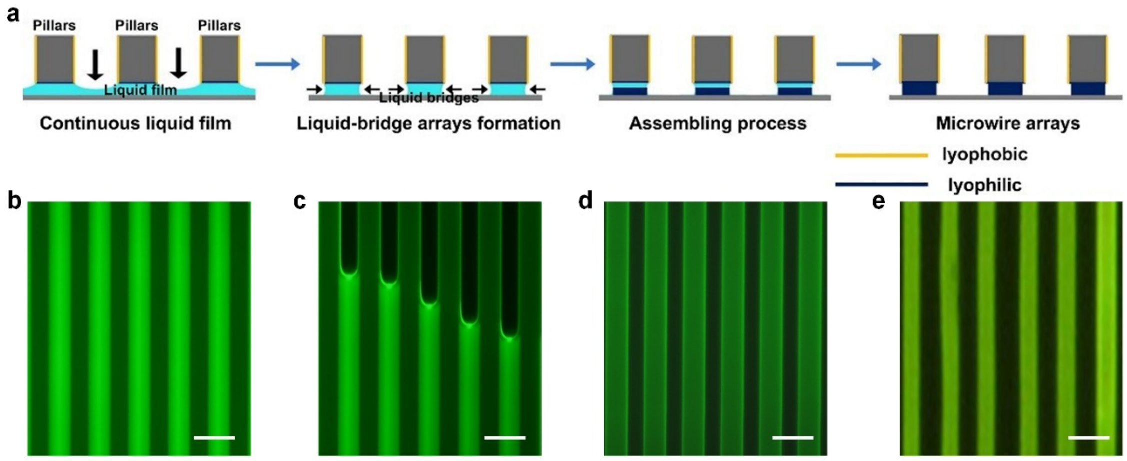

Supplementary Figure 3. Microwires assembling process. (a) Schematics of the microwires assembling process in the capillary-bridge-mediated method. A liquid thin film was first confined between an asymmetric-wettability micropillar-structured template and a flat target substrate followed by dewetting and formation of isolated capillary bridges anchored onto micropillars, generating 1D microwires arrays onto the target substrate. (b-e) *In situ* fluorescent microscopy observation showing the formation of capillary bridges and ordered deposition of 1D polymer/AIE arrays in the sandwich system. The assembling process starts with a continuous liquid film sandwiched between the silicon micropillar template and flat substrate. During solvent evaporation, controlled dewetting between micropillars divides the liquid film into a series of fragments for the formation of capillary bridges. Capillary bridges pinned at the pillar tops induce fabrication of 1D polymer/AIE arrays in the dewetting and growth process. After total evaporation of solvents, 1D polymer/AIE arrays are generated onto the target substrate. Scale bars are all 10 μm.

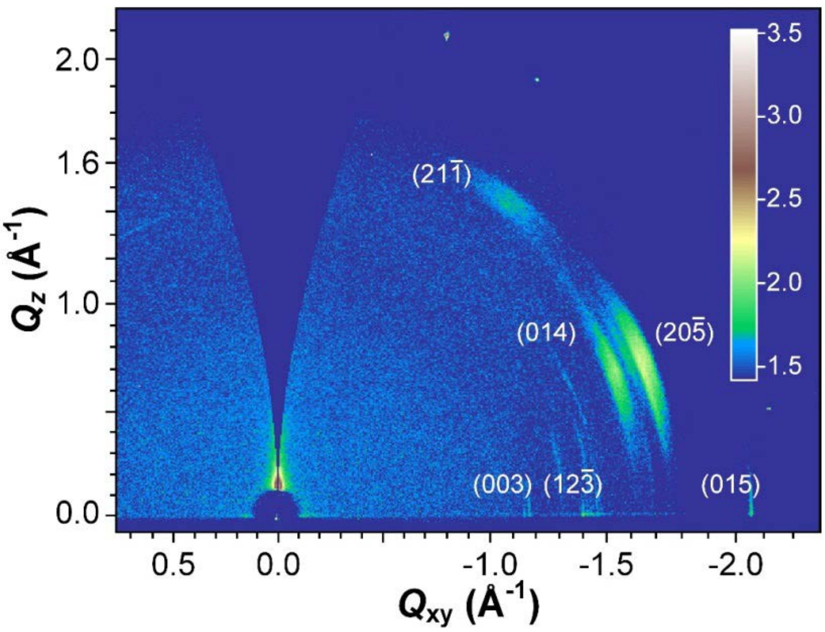

Supplementary Figure 4. GIWAXS pattern of 1D polymer arrays, illustrating their high crystallinity. The unit cell of PEO crystal has four helical molecules with parameters of a = 8.05 Å, b = 13.04 Å, c (fiber axis) = 19.48 Å and β = 125.4°, and the space group of $P2_1/a$.

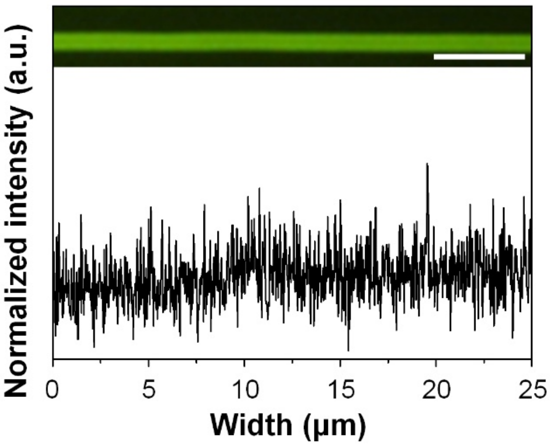

Supplementary Figure 5. Fluorescent intensity of 1D arrays obtained by the laser scanning confocal microscopy, illustrating the uniform distribution of AIE molecules in the 1D microstructures. Scale bar, 5 μm.

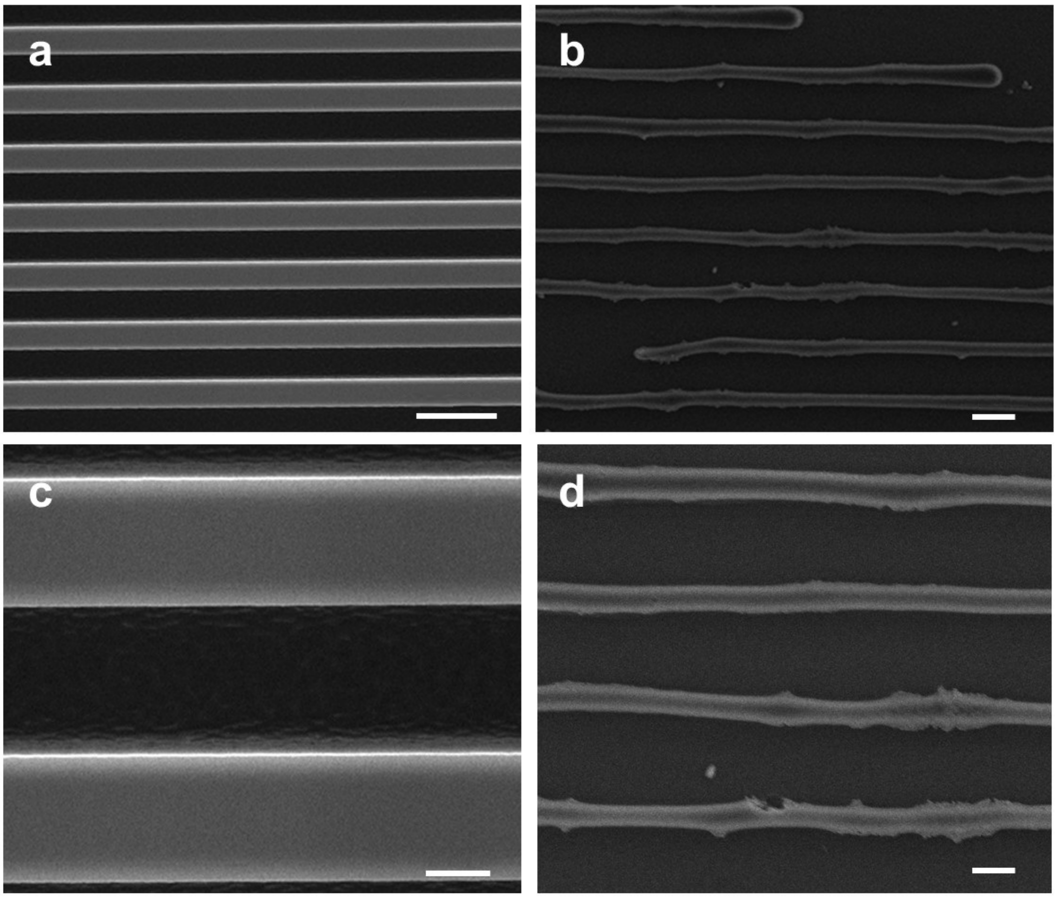

Supplementary Figure 6. Preparation of polymer/AIE molecules microwires regulated by the micropillar-structured template with different width and separation. (a) SEM image of the micropillar-structured template with the width of 2 μm, separation of 2 μm, and depth of 15 μm. (b) PES/$AnPh_3$ microwires array fabricated on quartz glass with the template of (a), indicating irregular and discontinuous microwires are formed. (c) SEM image of the micropillar-structured template with the width of 10 μm, separation of 10 μm, and depth of 15 μm. (d) PES/$AnPh_3$ microwires array fabricated on quartz glass with the template of (c), indicating irregular microwires are formed. Scale bar are all 5 μm.

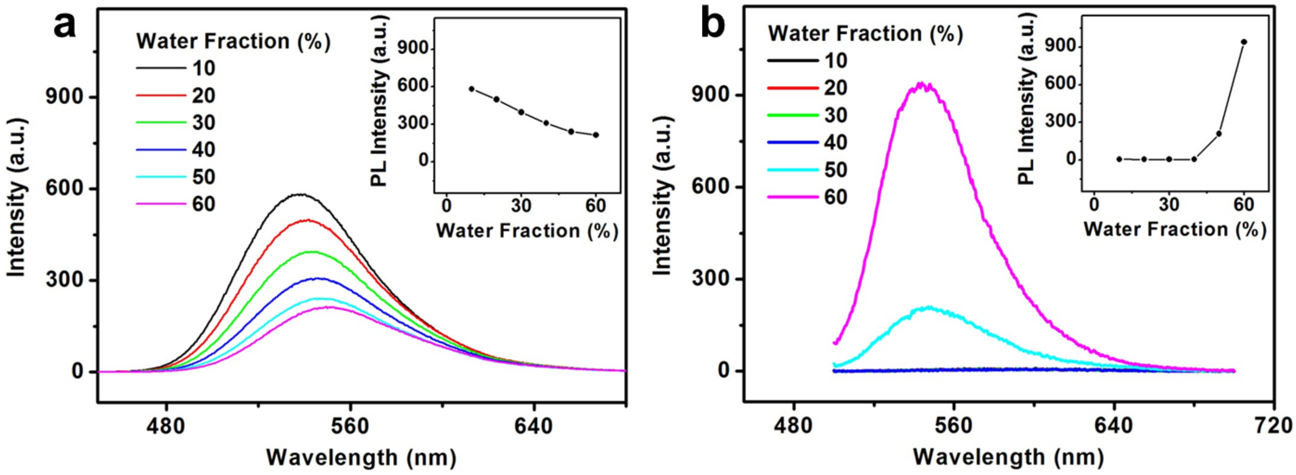

Supplementary Figure 7. Fluorescent characteristics of non-AIE molecule and AIE molecule. Fluorescent emission spectra of (a) Ph_3A_2 and (b) $AnPh_3$ in THF-water mixtures with different water fractions. The insets depict changes in fluorescent emission peak intensities under different water fractions. When the water fraction of THF-water mixtures increases from 10% to 60%, the fluorescent intensity of Ph_3A_2 shows a downward trend, yet the fluorescent intensity of $AnPh_3$ shows a 134-fold increase, demonstrating non-AIE feature of Ph_3A_2 molecule and obvious AIE feature of $AnPh_3$ molecule.

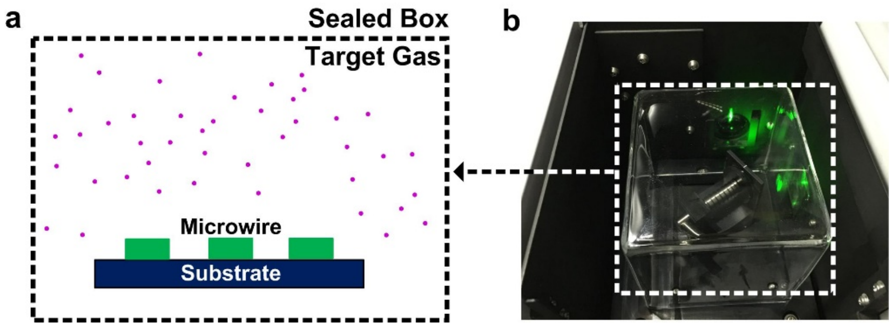

Supplementary Figure 8. Sensing performance measurement of microwires arrays. (a) Scheme and (b) digital photograph of measurement system, which was carried out in a sealed box, which was saturated with the target gas.

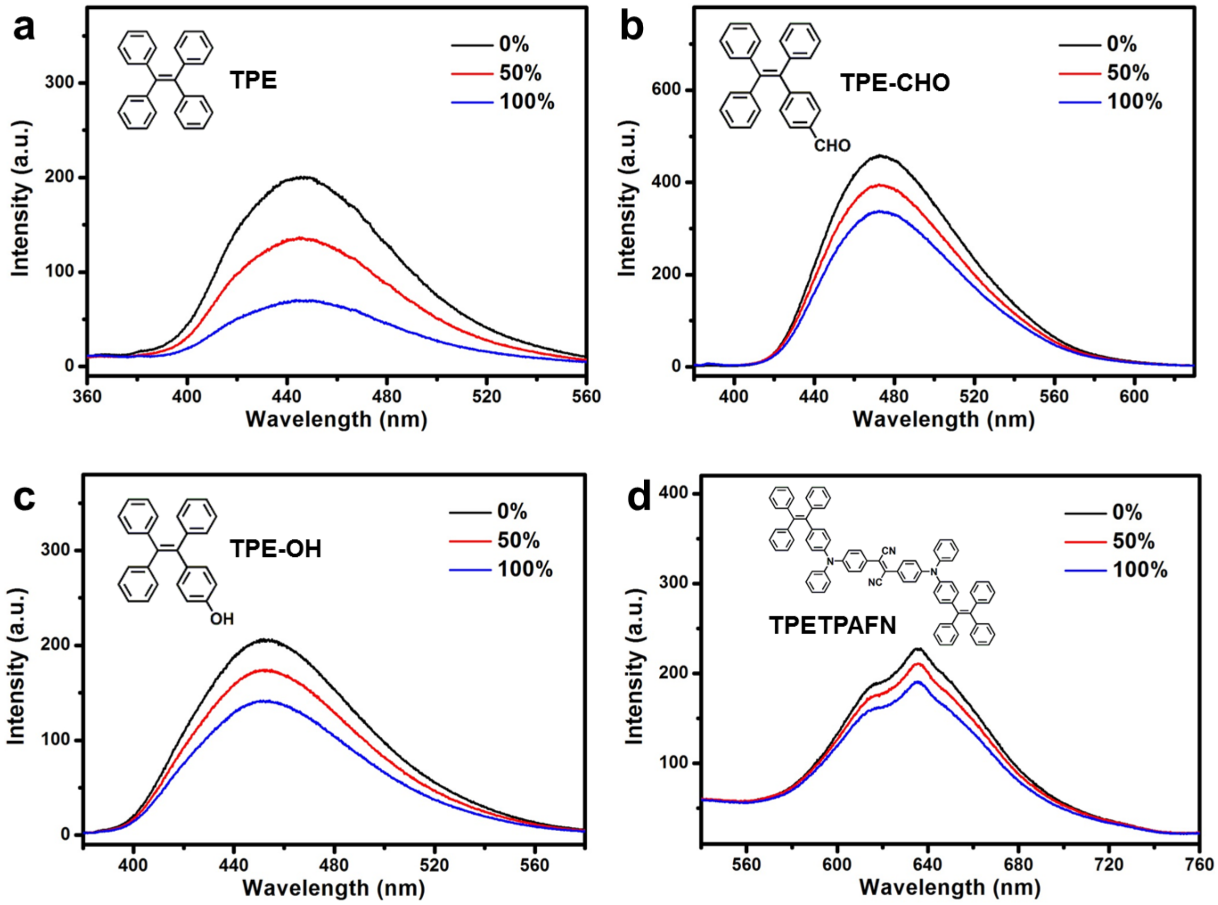

Supplementary Figure 9. Fluorescent emission spectra of PES/commercial AIE molecule microwires before (0%) and after (50% and 100%) exposure to acetone vapor. (a) TPE, (b) TPE-CHO, (c) TPE-OH, (d) TPETPAFN. 100% is the vapor pressure of acetone under ambient condition, and 50% is half of the vapor pressure of acetone under ambient condition. The molecular structures of the commercial AIE molecules are shown as the inset. The fluorescent excitation wavelengths are 340 nm, 350 nm, 350 nm, 490 nm for (a), (b), (c), (d), respectively.

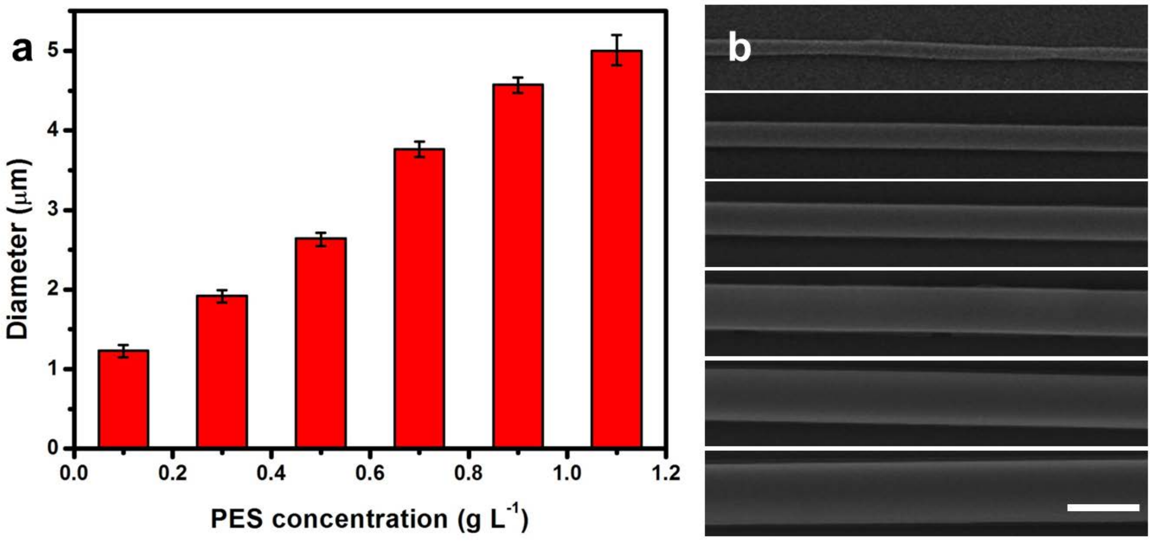

Supplementary Figure 10. The relationship of diameter of PES/$AnPh_3$ microwires and PES concentration. (a) The PES concentration dependence of diameter of PES/$AnPh_3$ microwires, shows an increasing trend of diameter as the PES concentration increases. Error bars, s.d. (n = 50) (b) SEM images of PES/$AnPh_3$ microwires in different PES concentrations (0.1, 0.3, 0.5, 0.7, 0.9 and 1.1 g L^{-1}, respectively). Scale bar, 5 μm.

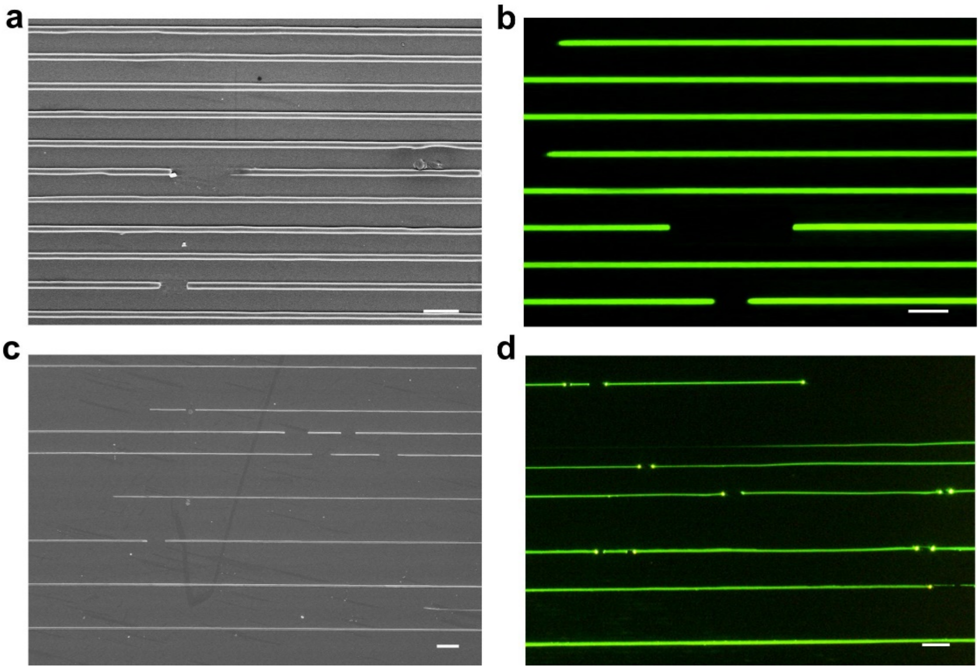

Supplementary Figure 11. PES/$AnPh_3$ microwires array fabricated on quartz glass. SEM images of PES/$AnPh_3$ microwires with different diameters: (a) *ca.* 1.23 µm, (c) *ca.* 0.55 µm. Fluorescent images of PES/$AnPh_3$ microwires with different diameters: (b) *ca.* 1.23 µm, (d) *ca.* 0.55 µm. Scale bars are all 5 µm.

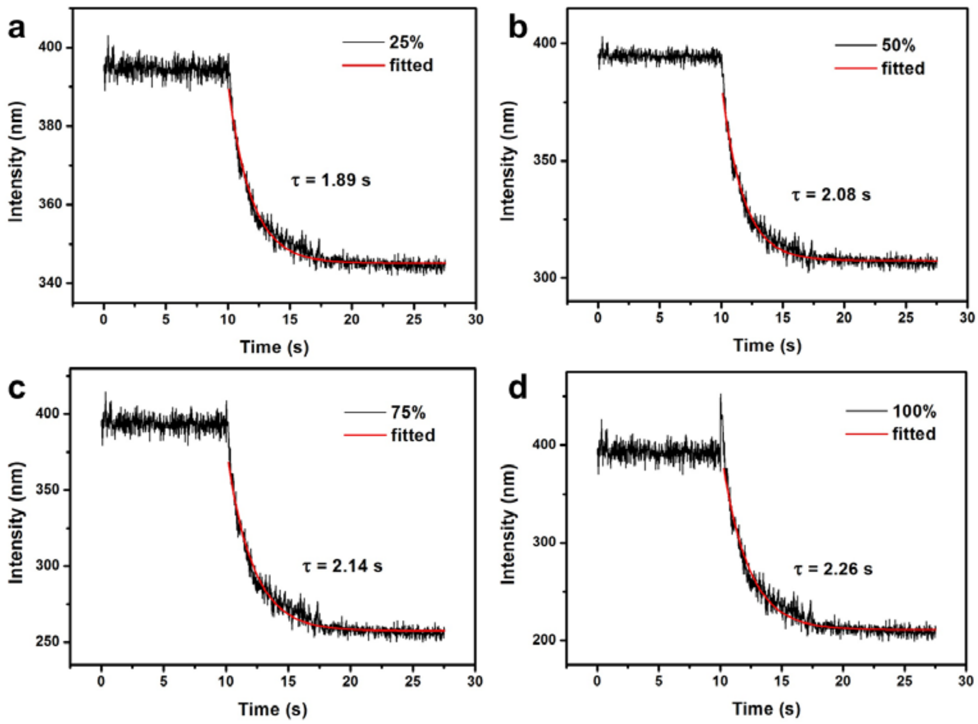

Supplementary Figure 12. Time-course of fluorescence quenching of the PES/$AnPh_3$ microwires upon exposure to acetone vapor at different concentration ((a) 25%, (b) 50%, (c) 75%, (d) 100%). The result indicates the sensor response times are 1.89 s, 2.08 s, 2.14 s, and 2.26 s for 25%, 50%, 75%, and 100% of acetone vapor concentration, respectively. 100% is the saturated vapor pressure of acetone under ambient condition, and 25%, 50%, and 75% are different proportions of vapor pressure of acetone under ambient condition. The fluorescent emission is set as 540 nm.

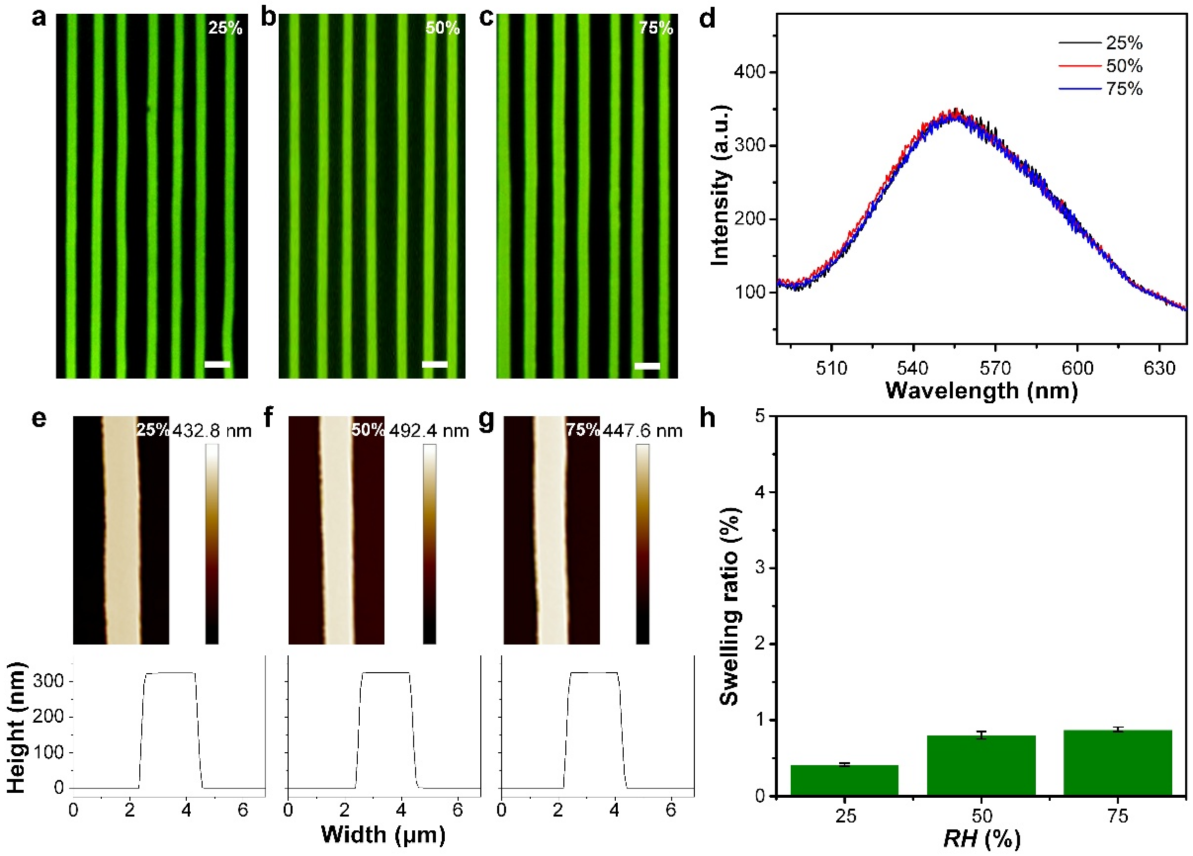

Supplementary Figure 13. The stability test of 1D array vapor sensors in the presence of water. (a-c) *In situ* top-view fluorescent microscopy observations and (d) fluorescent emission measurements of 1D arrays at *RH* of 25%, 50% and 75%, illustrating the stable photoluminescence in the presence of water. (e-g) AFM images of polymer/AIE microwires measured at *RH* of 25%, 50% and 75%. (h) The statistical swelling ratios of 1D arrays with less than 1% (0.42 ± 0.02%, 0.80 ± 0.05%, 0.87 ± 0.03%) at *RH* of 25%, 50% and 75%, demonstrating the stable morphology in the presence of water. Scale bars are all 5 μm. Error bars, s.d. (n = 10).

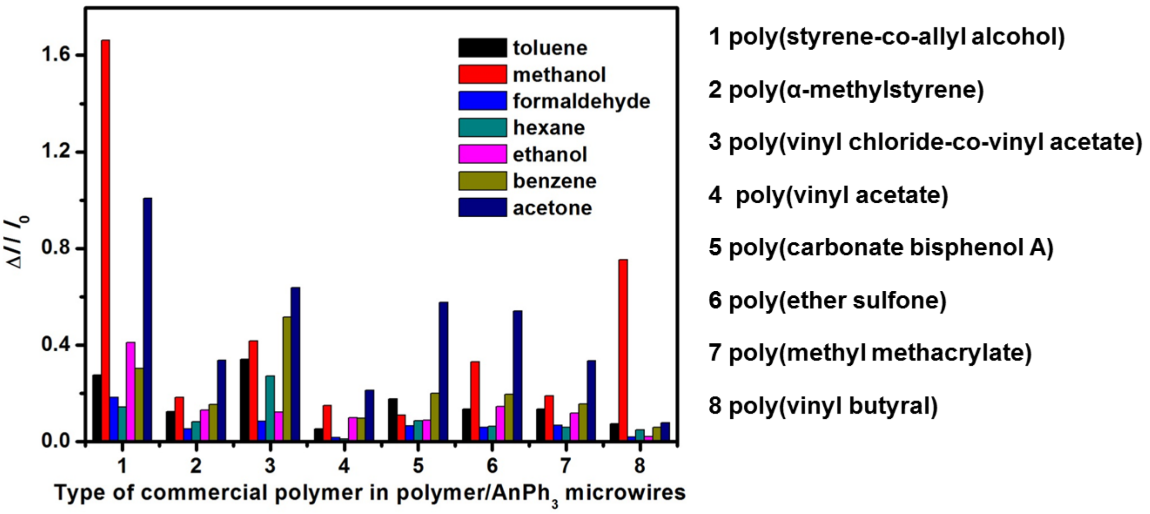

Supplementary Figure 14. Array-based vapor sensing of polymer/AIE molecules microwires array with commercial polymers. The fluorescent intensity variation data ($\Delta I/I_0$) obtained for arrays of eight polymer/$AnPh_3$ microwires during exposure to seven organic vapors. The used eight commercial polymers are poly(styrene-co-allyl alcohol), poly(α-methylstyrene), poly(vinyl chloride-co-vinyl acetate), poly(vinyl acetate), poly(carbonate bisphenol A), poly(ether sulfone), poly(methyl methacrylate) and poly(vinyl butyral). The seven organic vapors are toluene, methanol, formaldehyde, hexane, ethanol, benzene and acetone.

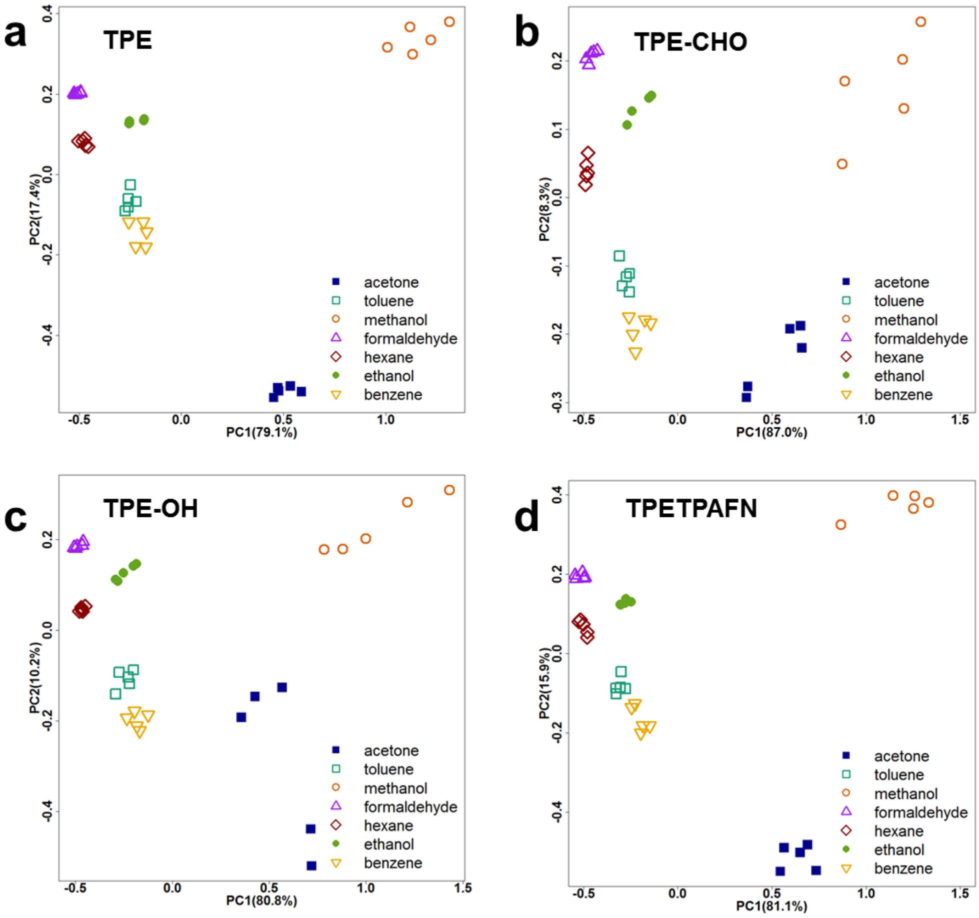

Supplementary Figure 15. PCA plot calculated from the fluorescent intensity variation data obtained for arrays of eight polymer/commercial AIE molecule microwires during exposure to seven organic vapors, as presented by the first two principal axes of PC1 and PC2. The used eight commercial polymers include poly(styrene-co-allyl alcohol), poly(α-methylstyrene), poly(vinyl chloride-co-vinyl acetate), poly(vinyl acetate), poly(carbonate bisphenol A), poly(ether sulfone), poly(methyl methacrylate), poly(vinyl butyral), and the commercial AIE molecules are (a) TPE, (b) TPE-CHO, (c) TPE-OH, (d) TPETPAFN. The PCA algorithm reveals the variance in the data can be captured by PC1 and PC2 are 96.5%, 95.3%, 91.0%, 97.0% for (a), (b), (c), (d), respectively, indicating that different types of the organic vapors are well separated.

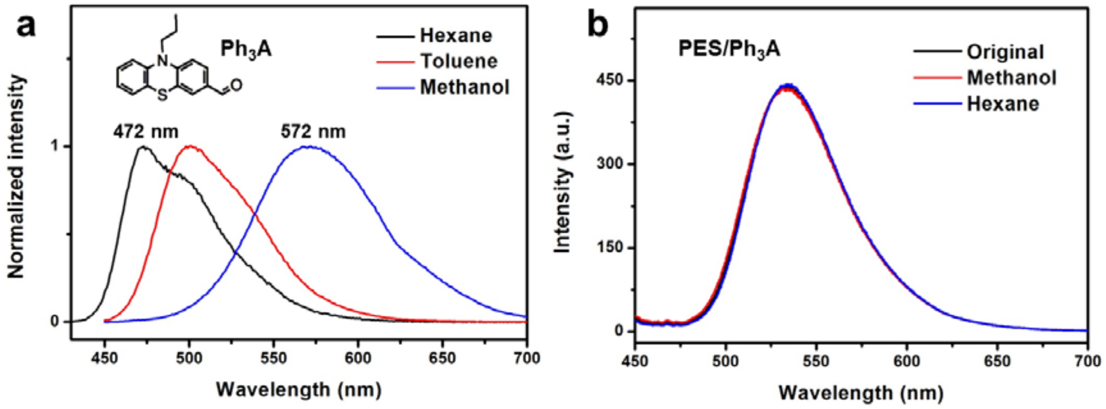

Supplementary Figure 16. (a) Normalized fluorescent emission spetra of a solvatochromic dye (Ph_3A) dissolved in three solvents (hexane, toluene, methanol) with different polarity, demonstrating large wavelength-shift (100 nm) from nonpolar solvent (hexane) to polar solvent (methanol). (b) Fluorescent emission spectra of PES/Ph_3A microwires before (original) and after exposure to saturated methanol or hexane vapor, indicating the wavelength-shift approach is not applicable to this polymeric swelling method.

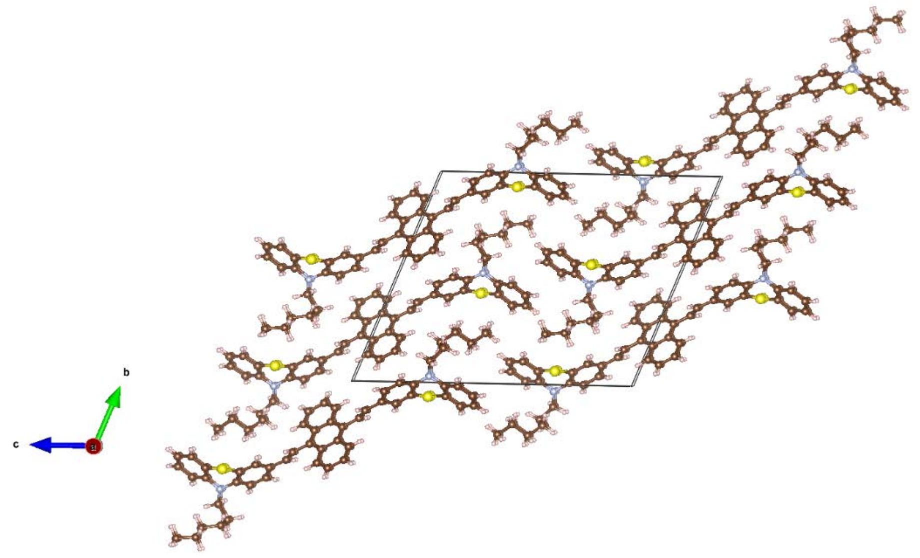

Supplementary Figure 17. Molecules packing in AnPh single-crystal structure from reference [1] (the yellow atom is S and gray is N).

	Top view	Side view
AIE/AIE1	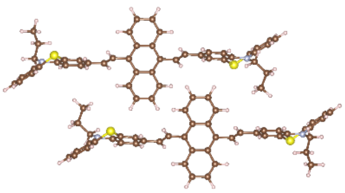	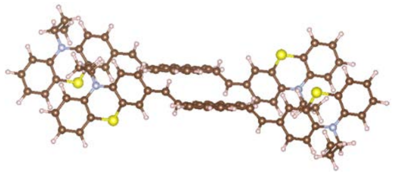
AIE/AIE2	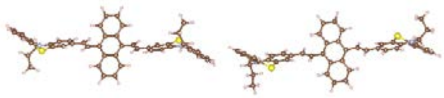	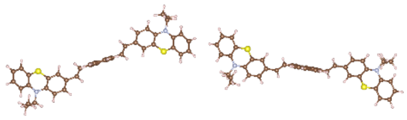
AIE/AIE3	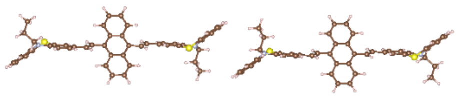	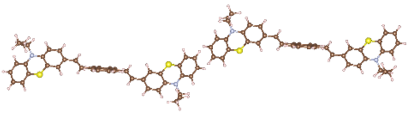
AIE/Polymer	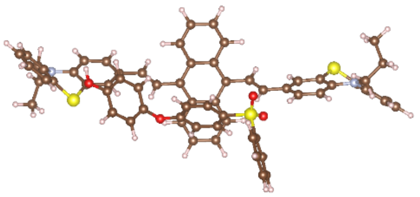	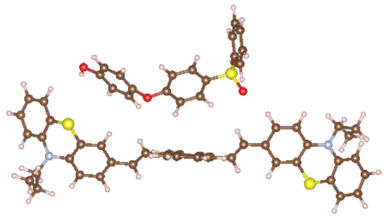

Supplementary Table 1. Optimized geometries structures of AIE dimers and AIE/Polymer (the red atom is oxygen) for DFT models.

Functional	$\triangle E_{AIE/AIE1}$ (eV)	$\triangle E_{AIE/AIE2}$ (eV)	$\triangle E_{AIE/AIE3}$ (eV)	$\triangle E_{AIE/Polymer}$ (eV)
wB97XD	-0.6868	-0.1226	-0.2122	-0.7452
B3LYP-D3(BJ)	-0.7333	-0.1240	-0.2374	-0.8388

Supplementary Table 2. The interaction energies of three AIE dimers and AIE/polymer with the alternative functional of wB97XD and B3LYP-D3(BJ).

Supplementary Note 1

High crystallinity of 1D polymer arrays. We employed GIWAXS to evaluate the quality and study the molecular packing of the 1D polymer (polyethylene oxide, PEO) arrays (Supplementary Fig. 4). The GIWAXS pattern shows the strong diffraction feature of 1D arrays, suggesting well-aligned and ordered arrangement of the polymer-chain assembly and measurable crystallinity. The scattering peaks of (003), ($12\bar{3}$), (014), ($21\bar{1}$), ($20\bar{5}$) and (015) can be identified by comparison with the reported research[2]. The unit cell of PEO crystal has four helical molecules with parameters of a = 8.05 Å, b = 13.04 Å, c (fiber axis) = 19.48 Å and β = 125.4°, and the space group of $P2_1/a$. This result evidences that the controlled dewetting process in the capillary-bridge-mediated method offers benefit to the fabrication of high-quality 1D arrays with ordered molecular packing and precise alignment.

Supplementary Note 2

Homodisperse of AIE molecules in the 1D polymer arrays. The high morphological quality of 1D polymer arrays is supposed to contribute to the uniform distribution of AIE dye molecules, which is a crucial factor of the high sensitivity of 1D array vapor sensors. The uniform fluorescent intensity of 1D arrays is certified by the laser scanning confocal microscopy, which indicates the homogeneous distribution of AIE molecules (Supplementary Fig. 5). In conclusion, the high quality of 1D polymer arrays assembled by our capillary-bridge-mediated system is beneficial for the high performance of 1D polymer/AIE molecule array vapor sensors.

Supplementary Note 3

Sensing performance measurement of microwires arrays. The vapor sensing measurements were carried out by placing the microwires arrays and a beaker with enough organic liquids into a sealed glass chamber. With the evaporation of analyte organic liquid, the sealed system is finally at the saturation vapor pressure of the organic molecules. After the 1D arrays sufficiently adsorb the organic vapor, a fluorescent spectrophotometer was employed for the optical measurements. All experiments were operated at room temperature and atmospheric pressure.

Supplementary Note 4

Patterning of 1D polymer arrays. First, dilute solutions were prepared by ultrasonic dissolution in organic solvent. Second, the polymer solution was carefully dropped onto the template and covered by a flat substrate, yielding a sandwich-shaped assembly system. Following the evaporation of solvent, the solution underwent unidirectional shrinkage, yielding strictly oriented capillary bridge arrays. These arranged capillary bridges provided isolated confined spaces for the assembly of polymer chains with strict orientation. Finally, high-quality 1D polymer arrays were successfully fabricated after the total evaporation of solvent.

Supplementary Methods

Materials. 1,4-Benzoquinone (98%) was purchased from Energy Chemical and used without further purifications. 3, 5-Dimethylaniline (98%), bis-(4-chlorophenyl)sulfone (98%), p-phenylenediamine (99%), 4-aminobenzoic acid (99%), 4-methylaniline (99%), 4-ethylaniline (98%), 4-butylaniline (97%), 1-bromopropane (99%) and phenothiazine (98%) were purchased from Sigma-Aldric and used without further purifications. Tetramethylene sulfone (99%) and heptadecafluorodecyl trimethoxysilane (97%) were purchased from J&K Scientific Ltd. and used without further purifications. Hydrochloric acid (AR), potassium carbonate (AR), Sodium nitrite (AR), Sodium bicarbonate (AR), zinc powder (AR), THF (AR), DMF (AR), phenol (AR), toluene (AR), methanol (AR), methanol (AR), hexane (AR), ethanol (AR), benzene (AR) and acetone (AR) were all purchased from Sinopharm and used without further purifications.

Synthesis of $AnPh_3$. Tetraethyl anthracene-9,10-diylbis(methylene) diphosphonate (0.24 g) and 10-propyl-10*H*-phenothiazine-3-carbaldehyde (0.32 g) were dissolved in THF (20 mL), and then t-BuOK (0.30 g) was added under N_2 gas. The solution was stirred at room temperature overnight. After removing the solvent under reduced pressure, the residue was recrystallized with THF/EtOH to give $AnPh_3$.

Synthesis of m-dimethylphenyl-containing PES. Deionized water (20 mL), ice and 3, 5-dimethylaniline (0.05mol) solution were added to a 1000 mL beaker equipped with a mechanical stirrer, a dropping funnel and a thermometer. Then 0.2 mol of hydrochloric acid was added to it, followed by dropwise adding of sodium nitrite (0.05 mol) aqueous solution. The mixture was stirred at 0-2 °C for 2 h. After filtration, the resulting solution was dropped into a stirring mixture of

1,4-benzoquinone (0.042 mol), sodium bicarbonate (0.134 mol) and water (100 mL) at 5-8 °C for 2 h, and then stirred at room temperature for another 2 h. The precipitate was collected by filtration, washed thoroughly with deionized water, and dried at ambient temperature. The obtained (3, 5-dimethyl)phenylbenzoquinone (0.05 mol), together with Zn powder (0.127 mol) and deionized water (60 mL) were added into a 100 mL three-necked flask, and heated to reflux with stirring. Meanwhile, 14.15 mL of HCl (11.8 mol L^{-1}) was dropwise added to it, and then refluxed for another 4 h. After filtration, the solid was precipitated from the filtrate. Pure (3, 5-dimethyl)phenylhydroquinone was obtained by recrystalization from toluene with the yield of 40%. ^{1}H NMR (400 MHz, d_6-DMSO) δ (ppm): 2.26 (s, 6H), 6.51-6.75 (m, 3H), 6.88 (s, 1H), 7.10 (s, 2H), 8.65 (s, 1H), 8.75 (s, 1H). (3, 5-dimethyl)phenylhydroquinone (5 mmol), bis-(4-chlorophenyl)sulfone (5 mmol), anhydrous K_2CO_3 (6 mmol), tetramethylene sulfone (TMS, 5.59 mL) and toluene (6 mL) were added into a 25 mL three-necked flask equipped with a mechanical stirrer, a nitrogen inlet with a thermometer and a Dean-Stark trap with a condenser. The system was allowed to reflux for 2-4 h and then the toluene was removed. The reaction mixture was heated to 170-180 °C. After 3 h, the polymerization was completed. The viscous solution obtained was then poured into water. The flexible threadlike polymer was pulverized into a powder with a blender. The polymeric powder was washed several times with hot water and ethanol and dried at 120 °C for 24 h, the yield was obtained as 90%. ^{1}H NMR (400 MHz, d_6-DMSO) δ (ppm): 2.25 (s, 6nH), 6.84-7.20 (m, 10nH), 7.65-8.00 (m, 4nH).

Synthesis of 4-aminophenyl-containing PES. The synthetic route of 4-aminophenyl-containing PES was similar to that of m-dimethylphenyl-containing PES, while the reactant of

p-phenylenediamine was used instead of 3, 5-dimethylaniline. The yield of intermediate of 4-aminophenyl hydroquinone was 35%. ^{1}H NMR (400 MHz, d_6-DMSO) δ (ppm): 5.10 (s, 2H), 6.35-6.75 (m, 5H), 7.10-7.30 (m, 2H), 8.5 (s, 1H), 8.65 (s, 1H). The yield of 4-aminophenyl-containing PES from 4-aminophenyl hydroquinone was obtained as 96%. ^{1}H NMR (400 MHz, d_6-DMSO) δ (ppm): 5.20 (s, 2nH), 6.40-6.58 (m, 2nH), 6.81-7.32 (m, 9nH), 7.72-8.11 (m, 4nH).

Synthesis of 4-carboxyphenyl-containing PES. The synthetic route of 4-carboxyphenyl-containing PES was similar to that of m-dimethylphenyl-containing PES, while the reactant of 4-aminobenzoic acid was used instead of 3, 5-dimethylaniline. The yield of intermediate of 4-carboxyphenyl hydroquinone was 36%. ^{1}H NMR (400 MHz, d_6-DMSO) δ (ppm): 6.54-6.88 (m, 3H), 7.58-8.05 (m, 4H), 8.66-9.10 (m, 2H), 12.85 (s, 1H). The yield of 4-carboxyphenyl-containing PES from 4-carboxylphenyl hydroquinone was obtained as 95%. ^{1}H NMR (400 MHz, d_6-DMSO) δ (ppm): 6.83-8.23 (m, 15nH), 12.90 (s, 1nH).

Synthesis of 4-methylphenyl-containing PES. The synthetic route of 4-methylphenyl-containing PES was similar to that of m-dimethylphenyl-containing PES, while the reactant of 4-methylaniline was used instead of 3, 5-dimethylaniline. The yield of intermediate of (4-methyl)phenylhydroquinone was 37%. ^{1}H NMR (400 MHz, d_6-DMSO) δ (ppm): 2.32 (s, 3H), 6.51-6.76 (m, 3H), 7.18 (d, 2H, J = 8.0 Hz), 7.40 (d, 2H, J = 8.0 Hz), 8.71 (s, 2H). The yield of 4-methylphenyl-containing PES from (4-methyl)phenylhydroquinone was obtained as 97%. ^{1}H NMR (400 MHz, d_6-DMSO) δ (ppm): 2.14 (s, 3nH), 6.84-7.39 (m, 11nH), 7.63-8.05 (m, 4nH).

Synthesis of 4-ethylphenyl-containing PES. The synthetic route of 4-ethylphenyl-containing PES was similar to that of m-dimethylphenyl-containing PES, while the reactant of 4-ethylaniline was used instead of 3, 5-dimethylaniline. The yield of intermediate of (4-ethylphenyl)phenylhydroquinone was 36%. ^{1}H NMR (400 MHz, d_6-DMSO) δ (ppm): 1.20 (t, 3H, J = 5.2 Hz), 2.52-2.70 (m, 2H), 6.51-6.76 (m, 3H), 7.20 (d, 2H, J = 8.0 Hz), 7.40 (d, 2H, J = 8.0 Hz), 8.73 (s, 1H), 8.77 (s, 1H). The yield of 4-ethylphenyl-containing PES from (4-ethylphenyl)phenylhydroquinone was obtained as 91%. ^{1}H NMR (400 MHz, d_6-DMSO) δ (ppm): 1.10-1.25 (m, 3nH), 2.51-2.72 (m, 2nH), 6.84-7.32 (m, 11nH), 7.68-7.95 (m, 4nH).

Synthesis of 4-butylphenyl-containing PES. The synthetic route of 4-butylphenyl-containing PES was similar to that of m-dimethylphenyl-containing PES, while the reactant of 4-butylaniline was used instead of 3, 5-dimethylaniline. The yield of intermediate of (4-butylphenyl)phenylhydroquinone was 35%. ^{1}H NMR (400 MHz, d_6-DMSO) δ (ppm): ^{1}H NMR (400 MHz, d_6-DMSO) δ (ppm): 0.90 (t, 3H, J = 5.2 Hz), 1.24-1.36 (m, 2H), 1.40-1.70 (m, 2H), 2.51-2.67 (m, 2H), 6.49-6.76 (m, 3H), 7.23 (d, 2H, J = 8.0 Hz), 7.42 (d, 2H, J = 8.0 Hz), 8.71 (s, 1H), 8.75 (s, 1H). The yield of 4-butylphenyl-containing PES from (4-butylphenyl)phenylhydroquinone was obtained as 95%. ^{1}H NMR (400 MHz, d_6-DMSO) δ (ppm): 0.86-1.00 (m, 3nH), 1.25-1.40 (m, 2nH), 1.42-1.65 (m, 2nH), 2.42-2.61 (m, 2nH), 6.84-7.32 (m, 11nH), 7.66-7.95 (m, 4nH).

Fabrication and selective modification of micropillar templates. Microgroove structure silicon pillar template was prepared by following steps. Silicon wafers (10 cm diameter, N doped, <100> oriented, 525 μm thick) were first structured by a direct laser writing apparatus (Heidelberg DWL200) that transferred the computer predefined design on the photoresist (Shipley Microposit S1800 series)

coated wafer with about 1 μm precision. After irradiation and development, the wafers were etched using deep reactive ion etching (DRIE, Alcatel 601E) with fluorine based reagents, for different times (10 s to 6 min) depending on the desired height of the structures. Grooved-pillar-structured silicon substrates with pillar gaps of 5μm, widths ranging from 5 μm, and heights of 20 μm were fabricated. After resist stripping (Microposit Remover 1165), the substrates were cleaned by ethanol and acetone prior to use. To selectively modify the sidewall and gap regions of micropillars, the micropillar's tops were firstly protected by a SU-8 layer. In detail, a SU-8 thin film was spin coated onto a flat silicon substrate followed by pressing onto the micropillar-structured template (Supplementary Fig. 1a). A SU-8 thin layer was coated onto micropillars after peeling off the flat substrate and baking at 95°C for 3 minutes (Supplementary Fig. 1b, c). Then, heptadecafluorodecyltrimethoxysilane (FAS) molecules with low surface energy were modified the top-protected templates (Supplementary Fig. 1d). After removing the SU-8 protection layer, micropillar templates with lyophilic tops and lyophobic sidewalls was fabricated (Supplementary Fig. 1e).

We employed SEM observation for the morphological characterization of the asymmetric-wettability micropillar template (Supplementary Fig. 2a, b). As shown in Supplementary Fig. 2c, d, a low contact angle of 24.6 ± 0.9° at the top and high contact angle of 112.6 ± 1.3° at the sidewall are exhibited. These results indicate the lyophilicity of micropillar tops and lyophobicity of micropillar sidewalls.

Supplementary References

[1] Zhang, X. et al. Piezofluorochromic properties and mechanism of an aggregation-induced emission enhancement compound containing *N*-hexyl-phenothiazine and anthracene moieties. *J. Phys. Chem. B* **115**, 7606-7611 (2011).

[2] Takahashi, Y. et al. Structural studies of polyethers, $(-(CH_2)_m-O-)_n$. X. Crystal structure of poly(ethylene oxide). *Macromolecules* **6**, 672-675 (1973)
